# In silico characterization, evolutionary analysis, and structural modeling of HSP70 gene family in carrot (*Daucus Carota L.)*

**DOI:** 10.1186/s12870-026-08102-y

**Published:** 2026-01-15

**Authors:** Maria Wali, Muhammad Sufyan Durrani, Muhammad Amjid, Asif Jan, Muhammad Maroof Khan, Mequanente Dagnaw

**Affiliations:** 1 Department of Biotechnology, Quaid-i-Azam University, Islamabad, Pakistan; 2 Biomedical engineering, Taiyuan University of Technology, Taiyuan, Shanxi China; 3 College of Pharmacy and Research Institute of Pharmaceutical Sciences, Gyeongsang National University, Jinju, Republic of Korea; 4 Saidu Group of Teaching Hospitals, Swat, Saidu Sharif, KP City 19200 Pakistan; 5 District Headquarter Hospital Charsadda, KP City, 24430 Pakistan; 6 Department of Pharmacy, University of Peshawar, KP City, 25000 Pakistan; 7 Department of Biotechnology, The University of Agriculture, PeshawarPeshawar, Pakistan; 8 Department of Epidemiology and Biostatistics, Institute of Public Health, University of Gondar, Gondar, Ethiopia; 9 Department of Medical Biotechnology, Institute of Biotechnology, University of Gondar, Gondar, Ethiopia

**Keywords:** Daucus carota, HSP70 gene family, Genome-wide analysis, Abiotic stress, Phylogenetic analysis, Segmental duplication, Homology modeling

## Abstract

Heat-shock protein 70 (HSP70) chaperones play indispensable roles in protein folding, homeostasis, and protecting plants against abiotic stress. While well-characterized in model species, a comprehensive genome-wide analysis of the HSP70 family in carrot (*Daucus carota L*.) has been lacking. The study aimed to identify the DCHSP70 gene family members in the *Daucus carota* genome and elucidate their evolutionary relationships, structural characteristics, and potential functional roles under stress conditions. We performed a systematic genome-wide analysis using bioinformatic approaches to identify family members. Phylogenetic relationships were analyzed using neighbor-joining methods, while chromosomal distribution and synteny were visualized to track evolutionary analysis. Promoter regions were screened for cis-regulatory elements, and protein-protein interaction (PPI) networks were constructed. Additionally, 3D structural modeling was performed using SWISS-MODEL and I-TASSER servers. A total of 52 DCHSP70 genes were identified and classified into six distinct phylogenetic subgroups based on their homology with *Arabidopsis thaliana* and *Solanum lycopersicum*. Chromosomal mapping revealed an uneven distribution across the nine chromosomes. Synteny analysis indicated that segmental duplication was the primary driving force behind the family evolution, with all the paralogous pairs undergoing strong purifying selection (Ka/Ks < 1). Analysis of cis-regulatory elements in the promoter regions identified a predominance of light, hormone, and stress-responsive motifs (e.g., ABRE, LTR, MBS), suggesting a complex regulatory network for environmental adaptation. The PPI network analysis revealed highly significant connectivity (*p* < 1.0e-16), with functional enrichment in protein refolding and cellular stress response. Structural modelling confirmed that most DCHSP70 proteins are conserved as monomers, with reliable 3D structures predicted even for divergent members like DCHSP70-15 and DCHSP70-46 using threading-based approaches. This study provides the first systematic characterization of the DCHSP70 family in carrot. The finding highlights the evolutionary conservation of these genes and their crucial role in the plant’s stress response mechanism, providing valuable targets for future breeding programs aimed at improving stress tolerance in *Daucus carota*.

**Supplementary Information:**

The online version contains supplementary material available at 10.1186/s12870-026-08102-y.

## Introduction

Light and temperature play a key role in determining the optimal growth and development of plants. However, unfavorable environmental conditions can have a detrimental impact on essential cellular activities [1]. Extreme temperatures disrupt cellular homeostasis, resulting in negative effects on both vegetative and reproductive growth stages. The crop yield and produce quality are compromised globally, along with other abiotic stresses [1, 2]. Increased temperature has a significant impact on essential metabolic processes, which include photosynthesis and respiration. These processes are affected by reduced enzymatic activity [3–5]. Heat stress impacts protein synthesis, folding, sorting, and translocation at the cellular level. It also changes the plasma membrane’s permeability, which temperature variations [6]. The heat stress response (HSR) is triggered by downstream signaling cascades that are initiated by changes in cell membrane fluidity and the unfolded protein response. The response involves the critical participation of heat shock factors (HSFs) and heat shock proteins (HSPs), also referred to as molecular chaperones. Transcriptomic studies on plants under heat stress have revealed that around 5% of genes are upregulated, with a considerable proportion of this response attributed to heat shock protein genes [7, 8]. These heat shock proteins (HSPs) are controlled by heat shock factors (HSFs) and are responsible for maintaining protein balance and other crucial functions in plants and other organisms, even under normal and stressful conditions [5, 7, 8]. Heat shock proteins (HSPs) are a group of proteins found in all types of cells, both prokaryotes and eukaryotes, first identified in *Drosophila melanogaster* in 1974 [9]. The HSPs are grouped into five main families according to their molecular weight: Hsp100, Hsp90, Hsp70, Hsp60, and small HSPs. They are in various parts of the cell and are highly expressed during conditions of abiotic stress [10, 11]. HSPs bind to their substrate reversibly and facilitate protein folding in an ATP-dependent manner, leading to disaggregation and proteolysis of substrate proteins.

According to Young, Moarefi et al. 2001 [12] Hsp90 family proteins function as dimers. They assist in protein sorting, cell signaling, and maintaining the conformation of receptors. Additionally, they interact with mutated proteins to keep them silent during normal conditions, forming a mutation buffering system in plants [13, 14]. Hsp100/Clp consists of two classes. Classification is based on the presence of nucleotide-binding domain numbers. Hsp100/Clp belongs to one of the superfamilies of the AAA + ATPase [15]. They regulate the disaggregation and degradation of misfolded proteins and collaborate with Hsp70 to remodel aberrant proteins [16, 17]. Members of the Hsp70 family of proteins are highly conserved molecular chaperones, characterized by two major domains and two subdomains [18, 19]. Hsp70 is the most abundant chaperone family in eukaryotes and assists in refolding, sorting, and translocation [20, 21], and proteolytic degradation of misfolded proteins. It also regulates the activity of biologically active forms of other proteins, such as HSFs, under both ideal and stressed environments [22].

Carrots, scientifically known as *Daucus carota*, are a popular root vegetable that belongs to the Apiaceae family [23]. In the US alone, they are widely cultivated around the world, with an estimated global crop value of $758 million. Carrots were originally introduced to North America as a medicinal herb but are now primarily grown as a food crop due to their high nutritional value and versatility in culinary applications. Till today, studies have been conducted to identify the members of the HSP family in *Arabidopsis thaliana* [24], barley [25], common bean [26], purple false brome (*Brachypodium distachyon)* [27], Cotton [28], *Soybean (Glycine max L.)* [29], tomato *(Solanum lycopersicum)* [3], Maize *(Zea mays)* [30], potato *(Solanum tuberosum L.)* [31], Pumpkin *(Cucurbita moschata)* [32] rice [33], *Theobroma cacao* [34], tobacco *(Nicotiana tabacum)* [35]. However, no literature was on the heat-shocked protein 70 family of carrots.

In this study, we employed different bioinformatic tools to identify 52 HSP70 family members throughout the entire carrot genome. We also studied chromosomal locations, gene duplication events, evolutionary history, gene structure, protein structure, and promoter elements associated with each identified HSP70 gene.

## Materials and methods

### Identification and nomenclature of DCHSP70 genes

The *Arabidopsis thaliana* HSP70 protein sequence (Accession: NP_187864.1) was utilized as a reference query to identify the HSP70 family from the *Daucus carota* genome. Sequence retrieval was performed using the Phytozome database (https://phytozome-next.jgi.doe.gov/). To ensure the reliability of the candidates, all retrieved sequences were subjected to domain analysis using an online motif finder. Sequences lacking the conserved HSP70 domain were excluded from the study. Finally, the validated genes were renamed systematically according to their chromosomal location in *D. carota.*

### Protein sequence analysis

The fundamental characteristics of the DCHSP70 genes, including chromosomal coordinates (start and end points), strand orientation, coding sequence length, and protein length, were retrieved from the plant database phytozome: (https://phytozome-next.jgi.doe.gov/;) [36]. Subsequently, physicochemical parameters such as molecular weight (MW), theoretical isoelectric point (pI), and the grand average of hydropathicity (GRAVY) were calculated using the ExPASy ProtParam tool (https://web.expasy.org/protparam/) [37].

### Subcellular localization

To predict the specific intracellular location of each DCHSP70 protein, the CELLO Life (http://cello.life.nctu.edu.tw/) and Wolf Psort (https://wolfpsort.hgc.jp/) servers were utilized. The predicted localization data derived from Wolf Psort was visualized as a heatmap using the TBtool software [38].

### Phylogenetic analysis

To elucidate the evolutionary relationships of the DCHSP70 gene family, a phylogenetic analysis was conducted using Molecular Evolutionary Genetics Analysis (MEGA) software (Version 12). Subsequently, a phylogenetic tree was constructed using the Neighbor-Joining method. To ensure the reliability of the tree topology, a bootstrap analysis was performed with 1,000 replicates [38]. Finally, the resulting tree file in Newick format was imported into the Interactive Tree of Life (ITOL) (https://itol.embl.de/) online tool for visualization and annotation. Using iTOL, the phylogenetic tree was customized to distinguish different groups by color, and the color of the gene names was changed for identification [39].

### Gene Structure, conserved Domain, and motif analysis

To visualize the structural diversity of the DCHSP70 gene family, a combined analysis of the phylogenetic tree, gene structure, and conserved protein features was performed. First, the phylogenetic tree was constructed using the Neighbor-Joining method in MEGA (Version 12), and a resulting tree file in Newick format was generated.

Conserved functional domains were predicted using the NCBI Conserved Domain Search (CDD) tool: (https://www.ncbi.nlm.nih.gov/Structure/cdd/wrpsb.cgi) [40]. Conserved protein motifs were identified using MEME Suite (http://memesuite.org/) [41], with the parameters set to identify a maximum of 10 motifs. Additionally, the exon-intron organization of the DCHSP70 genes was analysed by retrieving the corresponding GFF3 annotation file from the phytozome: (https://phytozome-next.jgi.doe.gov/;) plant database.

### Localization of chromosome

The chromosomal distribution of the DCHSP70 genes was visualized using the “Gene Location Visualization from GTF/GFF” module in TBtool. The genome annotation file (GFF3) and genome sequence data for D. carota were retrieved from the Phytozome database. To construct the physical map, the following input files were prepared. A genome ID list containing the specific transcript IDs of the 52 DCHSP70 members. File containing the original IDs and the given names assigned to them. Color map file containing the color code for genes on chromosomes, organized by their phylogenetic grouping, making it easy to visualize evolutionary relationships. The resulting diagram displayed the DCHSP70 gene mapped to its respective chromosomal coordinates.

### Gene duplication analysis

To investigate the evolutionary expansion and syntenic relationships of the DCHSP70 gene family, a circular ideogram was constructed using the Advanced Circus modules in TBtools. The chromosomal length data and gene coordinate information were extracted from the *D. carota* genome annotation files (GFFs) retrieved from the Phytozome database.

### Synteny analysis

To visualize gene duplication events and syntenic relations, the Genome FASTA and general feature format (GFF) annotation files for *Daucus carota* and *Arabidopsis thaliana* were retrieved from the Phytozome database. Collinear gene pairs and segmental duplication events were identified using the One Step MCScan tool. The syntenic relationships were then visualized using the Dual Synteny Plotter in TBtool [20].

### Evolutionary divergence

To assess the evolutionary selection pressure acting on the DCHSP70 paralogs, the non-synonymous (Ka) and synonymous (Ks) substitution rates were retrieved from the genome duplication database [26]. The (Ka/Ks) was calculated for each paralogous pair to determine the mode of selection: a ratio < 1 indicates purifying (negative) selection, > 1 indicates positive selection, and = 1 indicates neutral evolution [28]. Furthermore, the divergence time (T) for each duplicated gene pair was estimated using the formula: $\:\mathrm{T}=\:\frac{{Ks}}{{2r}}$ where r represents the synonymous substitution rate. For this study, a rate of *r* = 5.2 × 10^−9^ substitutions per synonymous site per year was adopted, based on the divergence rate reported for the Appiaceae family [42].

### Protein-Protein interaction (PPI) and gene ontology

To investigate the functional relationships and regulatory networks of the DCHSP70 gene family, a protein-protein interaction (PPI) network was constructed using the String database (v11.5) (https://string-db.org/). The amino acid sequences of the 52 DCHSP70 proteins were submitted as queries, with *Daucus carota* selected as the reference organism. The network was generated based on a minimum interaction score of 0.400 (medium confidence). The resulting PPI network was further visualized to identify key nodes and functional clusters [43].

To investigate the biological roles of the DCHSP70 family, the gene ontology (GO) enrichment analysis was performed directly within the STRING interface to classify the DCHSP70 gene based on biological process. The functional enrichment was considered statistically significant based on the False Discovery Rate, and the results were visualized to identify the predominant biological pathways associated with the gene family.

### Promoter region analysis

To investigate the transcriptional regulation of the DCHSP70 gene family, the promoter regions, defined as the 1,500 bp genomic sequences upstream of the translational initiation site (ATG), were retrieved from the *Daucus carota* genome database using Phytozome. The extracted sequences were submitted to the PlantCARE (plant Cis-Acting Regulatory Element) online database to predict and classify putative cis-acting regulatory elements. The identified elements were categorized based on their functional roles, including stress responsiveness [44].

### Homology modeling of proteins

The three-dimensional (3D) structures of the DCHSP70 proteins were predicted using the SWISS-MODEL server (https://swissmodel.expasy.org/). First, suitable templates were identified by searching the SWISS-MODEL template library (SMTL) using the BLAST and HHblits algorithms [45]. The quality of the generated models was assessed using the Global Model Quality Estimation (GMQE) and QMEAN scoring function. The oligomeric state was also predicted based on quaternary structure conservation, utilizing the QSQE score to estimate the reliability of interchain contacts [46]. However, for DCHSP70-15 and DCHSP70-46, the 3D structure was predicted using the I-TASSER (Iterative Threading ASSEmbly Refinement) server. This approach was employed to generate high-quality atomic models through multiple threading alignments and interactive structural assembly simulations. The final models for these two proteins were selected based on the confidence score (C-score) to ensure structural reliability [47].

## Results

### Identification and physicochemical analysis of DCHSP70 genes

A total of 52 unique, full-length HSP70 genes were identified in the *Daucus carota* genome using the *Arabidopsis* HSP70 sequence as a query. These genes were designated as DCHSP70-1 to DCHSP70-52 based on their chromosomal distribution (Supplementary File, Table 1). Detailed information regarding transcript IDs, chromosomal coordinates, strand orientation, coding sequences (CDS) length, and physicochemical properties, including protein length, molecular weight (MW), isoelectric point (pI), and grand average of hydropathicity (GRAVY), is listed in Supplementary File, Table 2.

The analysis revealed significant variation within the DCHSP70 family. The protein lengths ranged from 291 (DCHSP70-48) to 1,090 (DCHSP70-45) amino acids. Consequently, the molecular weights varied substantially, ranging from 32.58 kDa (DCHSP70-48) to 120.16 kDa (DCHSP70-45). The theoretical pI values indicated that while the majority of the DCHSP70 proteins are acidic, some members are basic, with values ranging from 4.96 (DCHSP70-11) to 9.27 (DCHSP70-28) (Supplementary File, Table 2).

### Subcellular localization

**Fig. 1:**
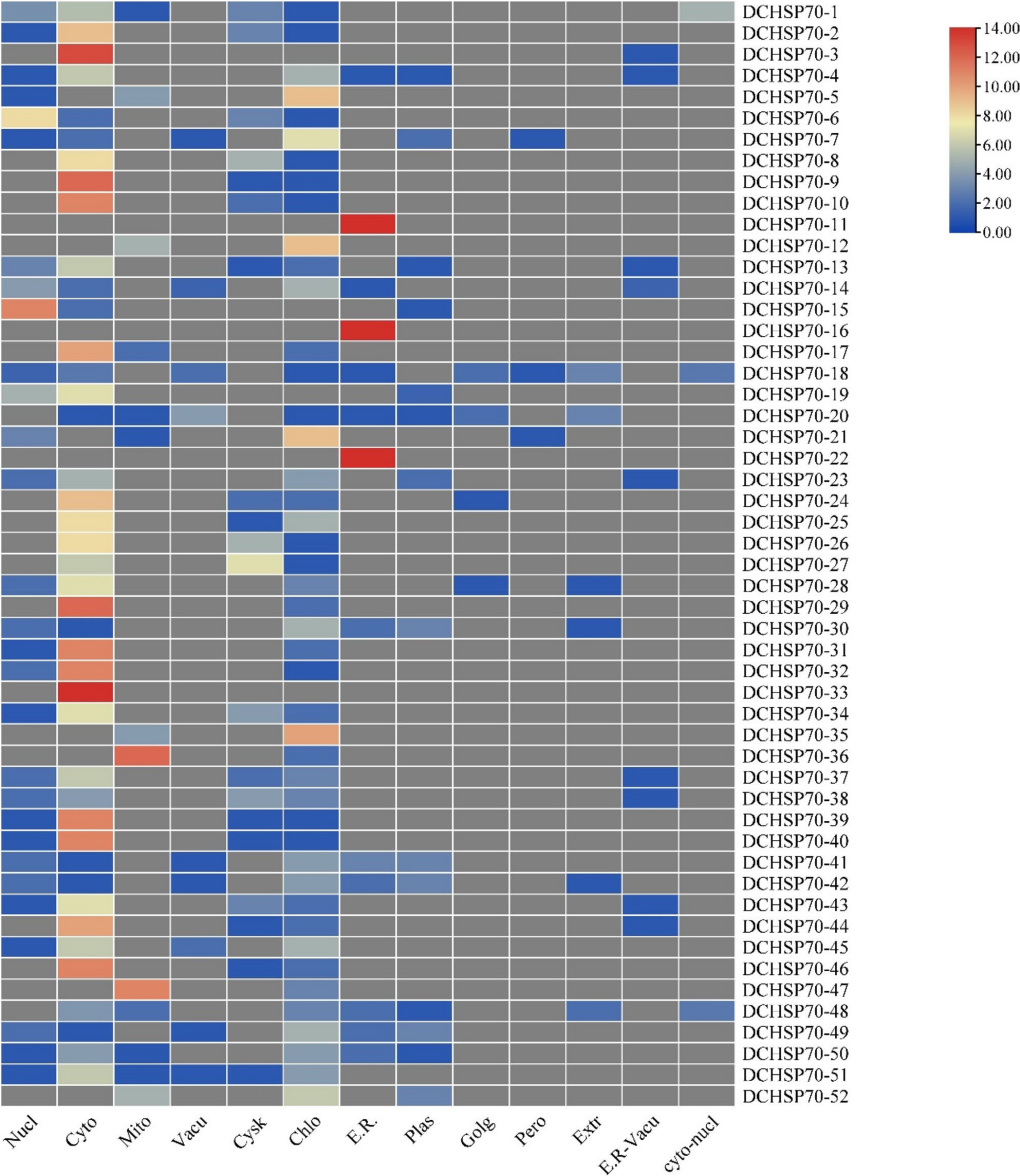
Subcellular localization prediction of the *Daucus carota* (DCHSP70) gene family. The corresponding heatmap displays the putative subcellular localization scores derived from the WoLF PSORT analysis. The color gradient represents the prediction confidence score, ranging from blue (low score) to red (high score). A high score indicated a strong probability of the protein localizing to that specific organelle. Abbreviations: Nucl: Nucleus, Cyto: Cytoplasm, Mito: Mitochondria, Vacu: Vacuole, Cysk: cytoskeleton, Chlo: Chloroplast, E.R.: Endoplasmic Reticulum, Plas: Plastid, Golg: Golgi Apparatus, Pero: Peroxisome, Extr: Extracellular

The subcellular localization analysis predicted that DCHSP70 proteins are distributed across various cellular organelles, with a distant bias towards the cytoplasm. Out of 52 identical DCHSP70 genes, 31 members (approximately 60% of the family) were predicted to be localized in the cytoplasm, consistent with their role as molecular chaperones. The chloroplast was the second most common localization site with 12 genes. Distinct localization signals were also identified for specific organelles. Three genes (DCHSP70-11, DCHSP70-16, and DCHSP70-22) showed strong and exclusive signals for Endoplasmic Reticulum localization. Two genes (DCHSP70-6 and DCHSP70-15) were predicted to localize primarily to the nucleus. Two genes (DCHSP70-36 and DCHSP70-47) were predicted to be mitochondrial. Signal genes were predicted to be localized to the cytoskeleton (DCHSP70-27) and the vacuole (DCHSP70-20) (Fig. 1).

The amino acid signals of each gene for different cell organelles were also predicted through the Wolf PSORT tool (Supplementary File, Table 3). This diverse subcellular distribution suggests that the DCHSP70 gene family has evolved to maintain protein homeostasis across multiple cellular compartments under normal and stress conditions.

### Analysis of the phylogenetic tree of HSP70s

A total of 78 HSP70 proteins from *Daucus carota*, *Arabidopsis thaliana*, and *Solanum lycopersicum* were used to construct the phylogenetic tree. The members were clustered into six groups (Groups 1–6). Group 1 was the largest, comprising 18 members. Group 4 and Group 6 were also substantial, containing 16 and 15 members respectively. The remaining groups were smaller: Group 3 (11 members), Group 5 (9 members), and Group 2 (8 members).

**Fig. 2:**
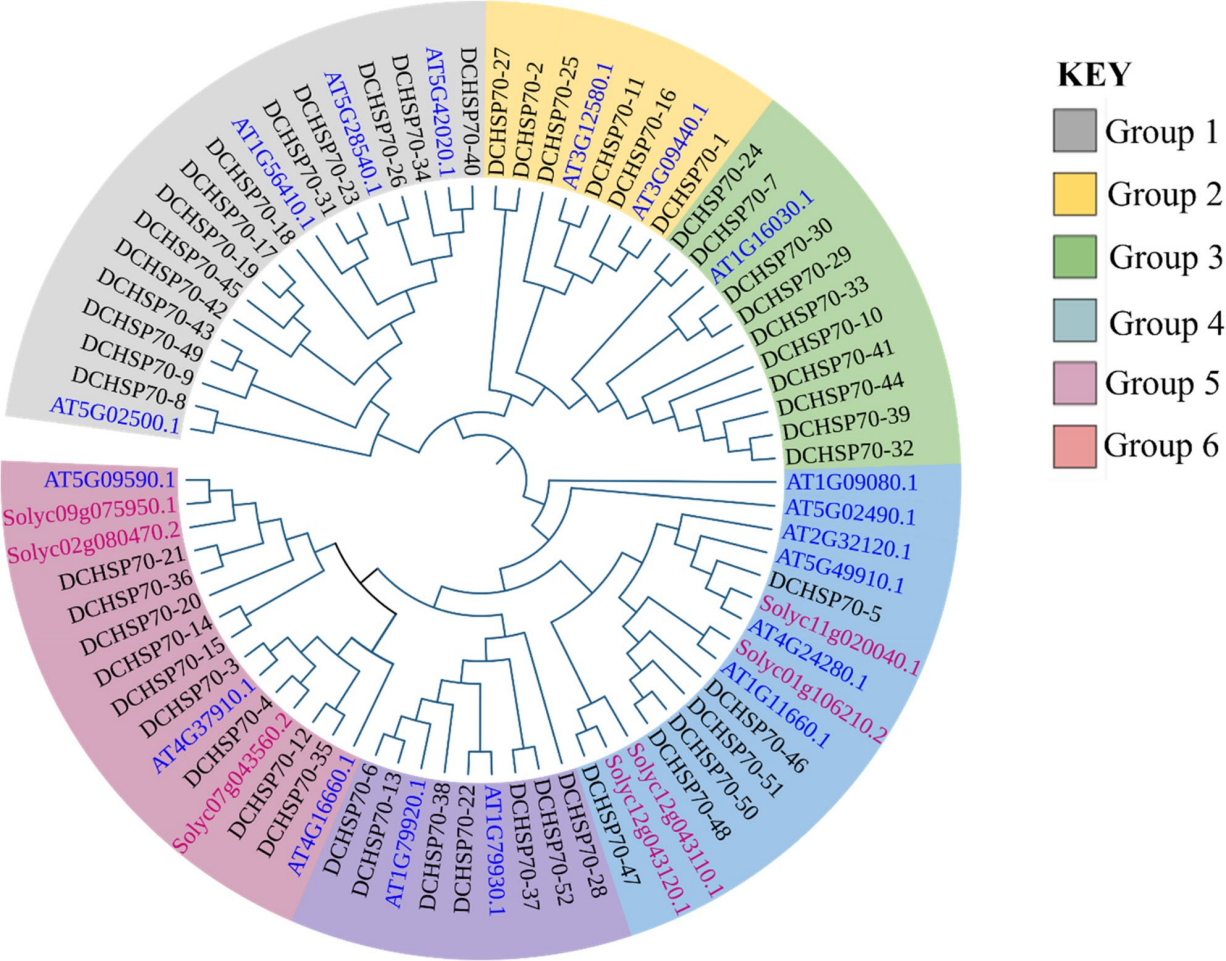
Phylogenetic analysis of the HSP70 gene family in *Daucus carota*. The tree shows the 52 D. carota (DCHSP70) proteins labelled as black, along with representative sequences from *Arabidopsis thaliana* (AT, blue labels) and *Solanum Lycopersicon* (Solyc, pink labels) to serve as references. The proteins are clustered into six distant subgroups (Group 1 to Group 6), distinguished by the colored background ring: Group 1 (grey), Group 2 (yellow), Group 3 (green), Group 4 (light blue), Group 5 (purple), and Group 6 (red)

Analysis of the tree showed 9 pairs of orthologous genes among the three species. The highest conservation was observed between *D. carota* and *A. thaliana*, which shared 5 orthologous pairs. Additionally, 2 pairs were identified between *D. carota* and *S. lycopersicum*, and 2 pairs were found between *A. thaliana* and *S. lycopersicum* (Fig. 2).

### Motifs analysis

**Fig. 3:**
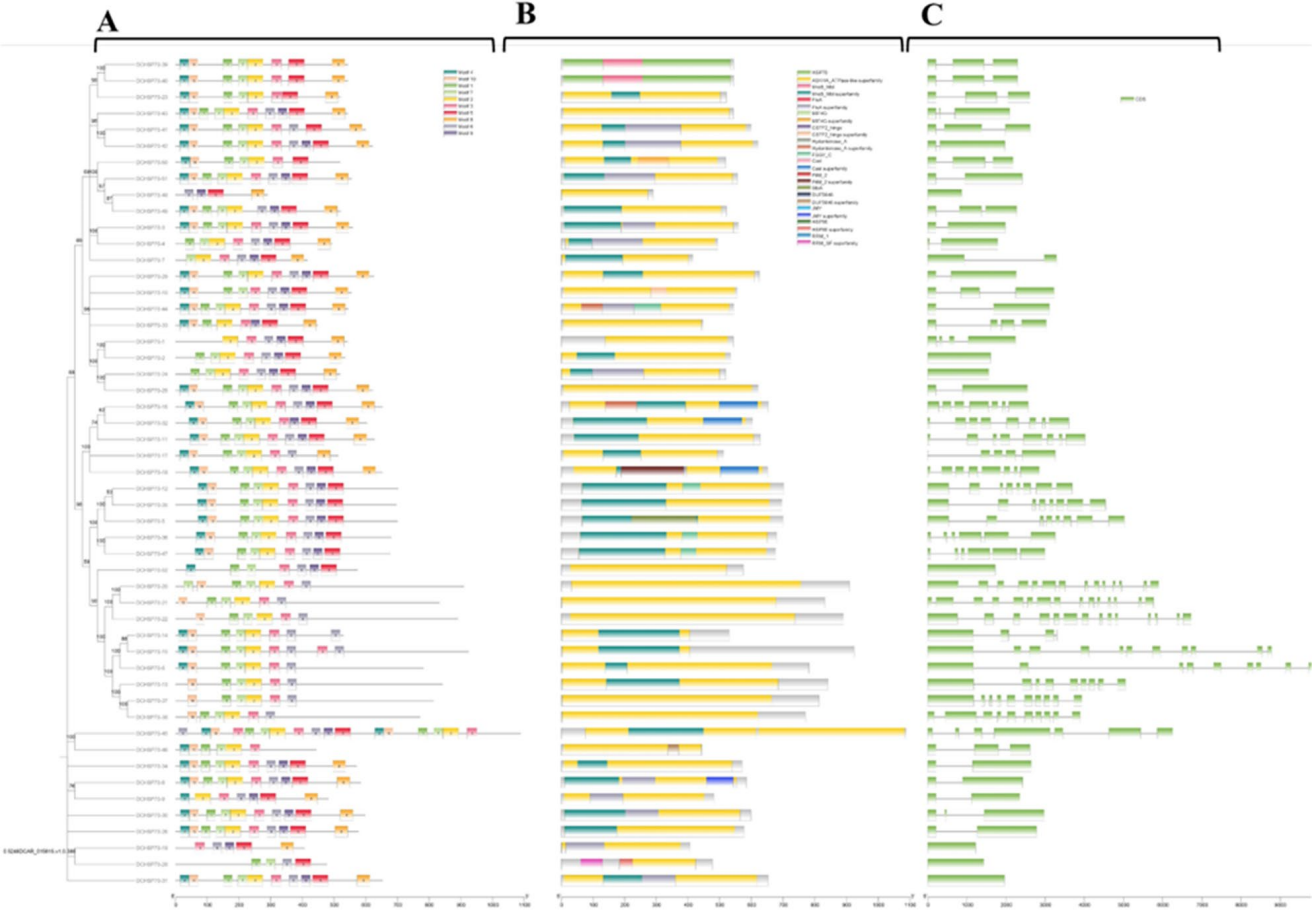
Structural and evolutionary analysis of the DCHSP70 gene family. The figure displays a combined view of the phylogenetic relationships and structural features of the 52DCHP70 genes. **A** The phylogenetic tree (left) was constructed using the neighbor-joining method. Adjacent to the tree is the distribution of conserved motifs (right), where different colored boxes represent specific motifs (Motif 1–10) identified by MEME analysis. **B** Conserved domain architecture, showing the position and span of the functional domain, e.g., the HSP70 domain, represented by colored bars. **C** Exon-intron structure of the DCHSP70 genes. Green boxes indicate exons (CDS), and the black lines represent introns

The MEME analysis revealed the presence of 10 distinct conserved motifs across the DCHP70 family members, designated as MEME-1 through MEME-10. The identified motifs exhibited variations in length, ranging from 29 to 50 amino acids (Table 1; Fig. 3A).

**Table 1:** Detailed information of the conserved motifs identified in DCHSP70 proteins

Motif No.	Motif Sequences	Width
MEME-1	YFNDSQRQATKDAATIAGLNVLRIINEPT	29
MEME-2	TAGDTHLGGEDFDNRLLNHFVEEFKRKHKKDISKNAKALRRLRNACEKAK	50
MEME-3	TRARFEELNMDLFRSCMEPVEKCLRDAGMDKS	32
MEME-4	TTYSCVGVWQHDRVEIIANDQGNRTTPSC	29
MEME-5	FSTASDDQTSVLIQVYEGERTRTKDNNLLGEFELSGIPPAPRGVPQIEVT	50
MEME-6	FNGKELCKSINPDEAVAYGAAVQAAILSG	29
MEME-7	KNVLIFDLGGGTFDVSLLKIKKGNFEVLA	29
MEME-8	KIEYAIKEAIEWLDANQSAEVEEYEYKKKELEAICNPIIPG	41
MEME-9	LLDVTPLSLGIETLGGVMTVIIPRNTTIP	29
MEME-10	VAFTDTERLIGDAAKNQAALNPENTIFDV	29

Motif 2 and Motif 5 were the largest, with a length of 50 amino acids. Motif 8 was the next largest with 41 amino acids, followed by Motif 3, which had 32 amino acids. The remaining six motifs, i.e., Motif 1, 4,6, 7, 9, and 10, were the shortest, all sharing a consistent length of 29 amino acids. Members belonging to the same phylogenetic group generally shared similar motif compositions, reinforcing their evolutionary relatedness.

### Analysis of conserved protein domain

The analysis of conserved domains confirmed the presence of the signature HSP70 superfamily domain in all 52 identified DCHSP70 proteins, validating their classification within the Heat Shocked Protein 70 family (Figure 3B). While the HSP70 domain was universally conserved, specific members exhibited unique domain architectures, suggesting functional diversification. DCHSP70-16 was found to uniquely contain the Hydantoinase_A superfamily domain. DCHSP70-28 exhibited a complex architecture containing both the HSP90 superfamily domain and the RRM_SF (RNA Recognition Motif) superfamily domain. DCHSP70-46 was the only member identified to possess the MIT CorA-like superfamily domain. The presence of these additional domains indicates that, in addition to their chaperone activities, these specific isoforms may play specialized roles in other cellular processes or stress signaling pathways.

### Gene structure analysis

The structural analysis diversity of the DCHSP70 genes was analyzed by examining their exon-intron organization (Fig. 3C). The number of exons varied significantly across the family, ranging from 1 to 15 exons, indicating a high degree of structural complexity. Four genes, DCHSP70-2, DCHSP70-6, DCHSP70-31, and DCHSP70-48, were identified as having no intron, containing only one exon and 0 introns. This lack of introns is a characteristic often observed in certain prokaryotic-derived or rapidly induced stress response genes. The remaining members contained multiple exons. Notably, DCHSP70-21 exhibited the most complex structure, containing the highest number of exons, i.e., 15 exons, and consequently the highest number of introns, i.e., 14 introns. Most of the genes exhibited a conserved structural pattern within their respective phylogenetic groups, supporting their evolutionary relationships. In this analysis, untranslated regions (UTRs) were not detected in the retrieved gene models. This may be attributed to the current genome annotation status or specific evolutionary divergence within the *Daucus carota* lineage.

### Chromosomal location

**Fig. 4:**
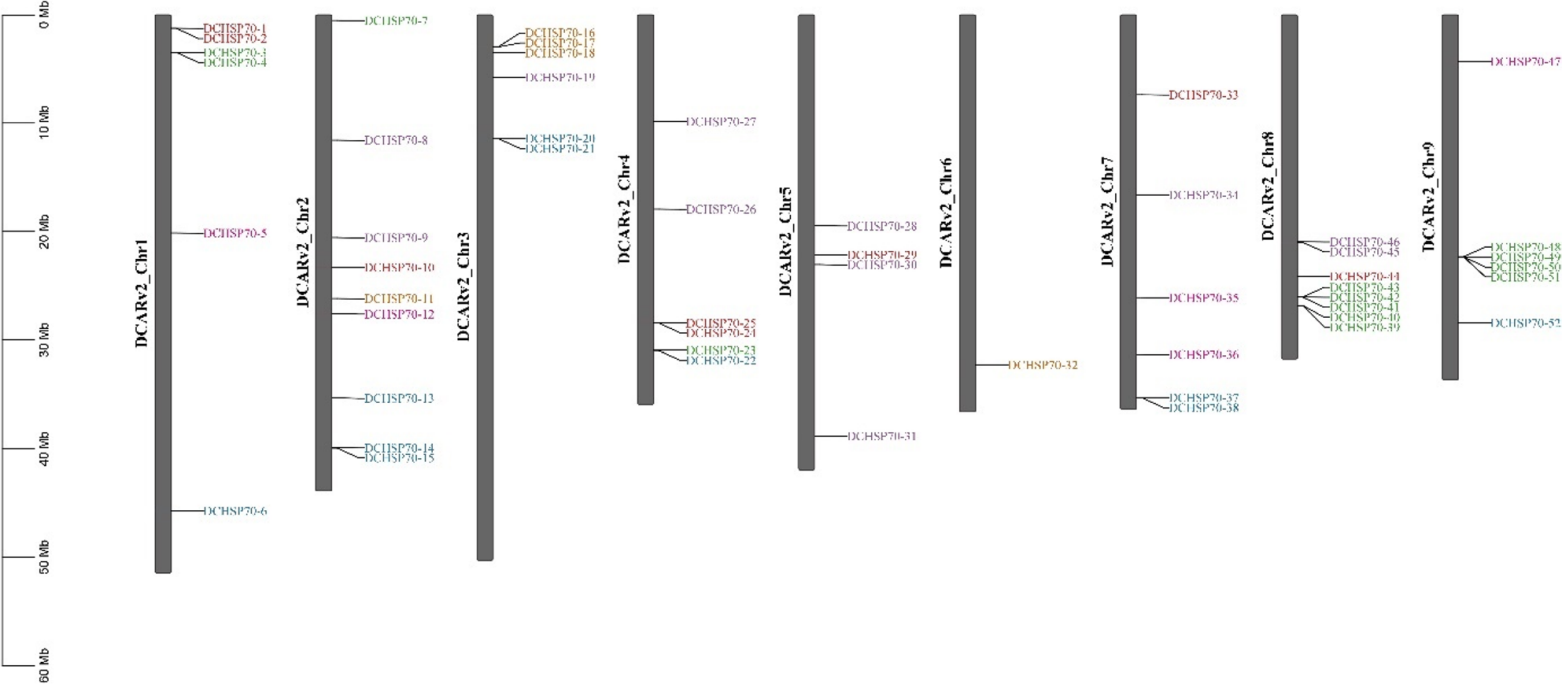
Chromosomal distribution of the 52 DCHSP70 genes on the 9 *Daucus carota* chromosomes. The vertical gray bars represent the nine chromosomes (DCARv2_Chr1 to DCARv2_Chr9). The scale on the left indicates the chromosomal length in megabases (Mb). The names of the DCHSP70 genes are listed on the right side of each chromosome at their respective physical locations. The gene labels are color-coded (e.g., red, green, blue) to match subfamilies defined in the phylogenetic analysis. High-density gene clusters are visible on the distal ends of Chromosomes 8 and 9

The chromosomal localization analysis revealed that the 52 DCHSP70 genes are unevenly distributed across the 9 chromosomes (Chr1-Chr9) of the *Daucus carota* genome (Fig. 4). The distribution destiny varies significantly among the chromosomes. Chromosomes 8 and 9 harbor the highest number of genes, appearing as densely populated hotspots. In contrast, Chromosome 5 and Chromosome 7 contain the fewest members, indicating a non-random distribution pattern across the genome. The genes on the map are color-coded to correspond with the phylogenetic groups identified in the phylogenetic tree. The presence of different colored genes on the same chromosome (e.g., Chromosome 1 containing red, green, and blue labels) indicates that the expansion of different phylogenetic groups occurred across multiple chromosomes rather than being restricted to a single locus.

### Gene duplication analysis

**Fig. 5:**
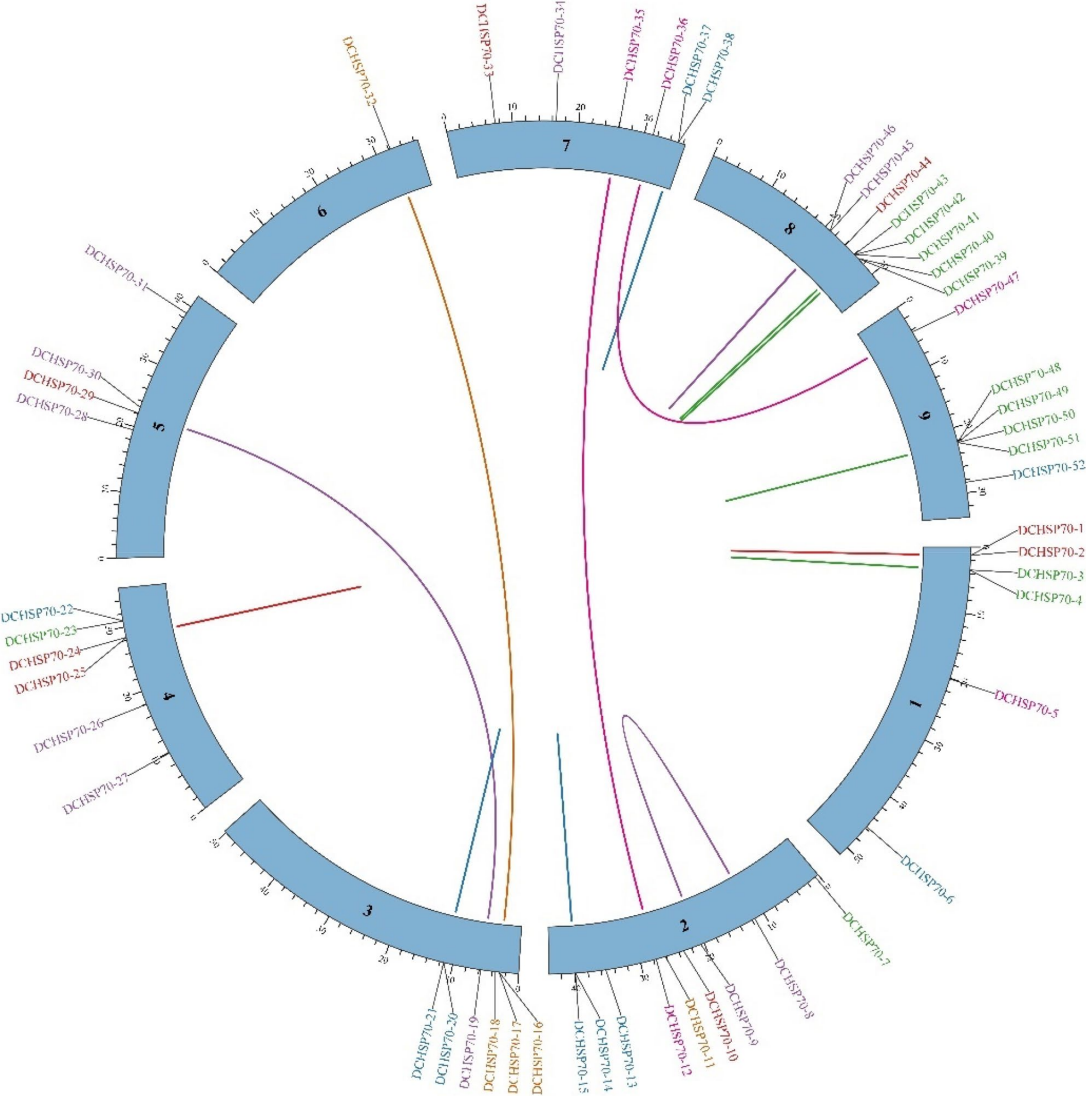
Circus ploy visualization of the DCHSP70 gene family in *Daucus carota*. The outer circle represents the 9 chromosomes, indicated by colored blocks. The location of all the 52 genes is marked on the chromosomes, and the color is coded according to their phylogenetic classification. The colored curved lines in the center show the segmentally and tandem duplicated gene pairs, highlighting the syntenic relationships and evolutionary expansion of the family

To investigate the evolutionary mechanism driving the expansion of the DCHSP70 gene family, a synteny analysis was performed using a Circus plot to visualize interchromosomal relationships (Fig. 5). The analysis revealed distant duplication events within the *Daucus carota* genome. For instance, syntenic pairs were observed between Chromosome 2 and Chromosome 7, connecting the DCHSP70-12 regions to the DCHSP70-35, DCHSP70-16, and 35 regions, between Chromosome 3 and Chromosome 6, as well as between Chromosome 1 and Chromosome 8. Tandem duplication also contributed to the local expansion of specific subfamilies, where multiple genes are physically adjacent to one another. Overall, the presence of these syntenic links confirms that segmental duplication has played a pivotal role in the evolutionary history and diversification of the DCHSP70 gene family in carrot.

### Synteny analysis

**Fig. 6:**
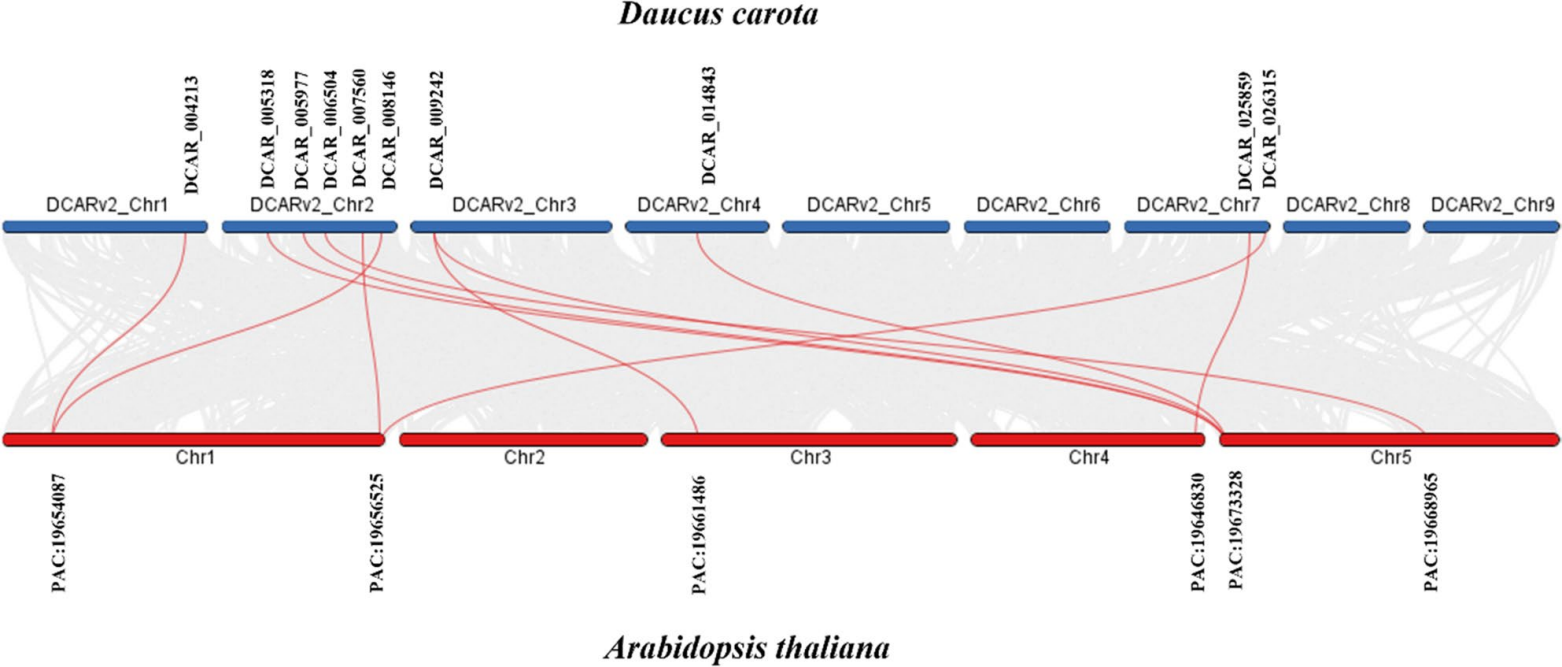
Synteny analysis of HSP70 genes between *Daucus carota* and *Arabidopsis thaliana*. The graphic illustrates the collinear relationships between the *Daucus carota* genome (top, blue bars) and the *Arabidopsis thaliana* genome (bottom, red bars)

Synteny analysis revealed the presence of two conserved regions/genes on the carrot chromosome. To explore the evolutionary origin and orthologous relationships of the DCHSP70 family, a comparative synteny analysis was performed between *Daucus carota* and the model plant. We identified an evolutionary relationship between *D. carota* genes and *Arabidopsis thaliana* (Fig. 6).

Within these syntenic blocks, specific orthologous gene pairs were identified between the DCHSP70 family and *Arabidopsis* HSP70 genes connected by the red lines e.g., DCHSP70-6, DCHSP70-8, DCHSP70-9, DCHSP70-11, DCHSP70-13, DCHSP70-14, DCHSP70-19, DCHSP70-19, DCHSP70-26, DCHSP70-36, and DCHSP70-37 with *Arabidopsis thaliana* genes PAC:19,654,087, PAC:19,673,328, PAC:19,673,328, PAC:19,668,965, PAC:19,656,525, PAC:19,654,087, PAC:19,661,486, PAC:19,673,328, PAC:19,673,328, PAC:19,646,830, and PAC:19,656,525 respectively. Dual synteny analysis of *D. carota* and *A. thaliana* revealed 11 ortholog genes (Table 2).

**Table 2:** List of orthologous HSP70 gene pairs between *Daucus Carota* and *Arabidopsis thaliana.* The table enumerates the specific orthologous pairs identified through comparative synteny analysis. It lists the chromosomal location and gene ID for each *D. Carota*

Chromo	Gene ID	Chromo	Gene ID
DCARv2_Chr1	DCAR_004213	Chr1	PAC:19,654,087
DCARv2_Chr7	DCAR_026315	Chr1	PAC:19,656,525
DCARv2_Chr7	DCAR_025859	Chr4	PAC:19,646,830
DCARv2_Chr3	DCAR_009242	Chr3	PAC:19,661,486
DCARv2_Chr3	DCAR_009242	Chr5	PAC:19,673,328
DCARv2_Chr2	DCAR_008146	Chr1	PAC:19,654,087
DCARv2_Chr2	DCAR_007560	Chr1	PAC:19,656,525
DCARv2_Chr2	DCAR_005977	Chr5	PAC:19,673,328
DCARv2_Chr2	DCAR_006504	Chr5	PAC:19,668,965
DCARv2_Chr2	DCAR_005318	Chr5	PAC:19,673,328
DCARv2_Chr4	DCAR_014843	Chr5	PAC:19,673,328

### Evolutionary divergence

To evaluate the evolutionary constraints acting on the DCHSP70 gene family, the non-synonymous (Ka) and synonymous (Ks) substitution rates were calculated for 14 identified paralogous pairs (Table 3). The Ka/Ks ratio is a key indicator of selection pressure, where a ratio less than 1 indicates purifying selection, a ratio equal to 1 suggests neutral evolution, and a ratio greater than 1 implies positive selection. In this study, the Ka/Ks ratios for all 14 paralogous pairs were significantly less than 1, ranging from 0.0257 (DCHSP70-8/DCHSP70-9) to 0.7986 (DCHSP70-1/DCHSP70-2). This consistent observation of Ka/Ks < 1 suggests that the DCHSP70 gene family has undergone strong purifying selection during its evolution, indicating that deleterious mutations were actively eliminated to maintain the conserved structure and function of these proteins. The divergence times estimated from the Ks values indicate that the duplication events occurred over a broad evolutionary timeframe. The estimated divergence ranged from 0.64 million years ago (MYA) for the recent pair (DCHSP70-20/DCHSP70-21) to 33.51 MYA for the most ancient pair (DCHSP70-19/DCHSP70-29), suggesting a continuous expansion history for the DCHSP70 gene family in carrot [48].

**Table 3:** Estimated Ka/Ks ratios and divergence times for DCHSP70 paralogous gene pairs

Seq_1	Seq_2	Ka	Ks	Ka/Ks	Time (MYA)
DCHSP70-1	DCHSP70-2	0.1236	0.1548	0.7986	11.8893
DCHSP70-3	DCHSP70-4	0.0968	0.1691	0.5726	9.3087
DCHSP70-8	DCHSP70-9	0.0162	0.6311	0.0257	1.5585
DCHSP70-12	DCHSP70-35	0.0422	0.8539	0.0495	4.0608
DCHSP70-14	DCHSP70-15	0.0185	0.0356	0.5195	1.7796
DCHSP70-16	DCHSP70-32	0.3404	2.2122	0.1539	32.7355
DCHSP70-19	DCHSP70-29	0.3485	2.4411	0.1428	33.5111
DCHSP70-20	DCHSP70-21	0.0067	0.0147	0.4533	0.6406
DCHSP70-24	DCHSP70-25	0.0521	0.1054	0.4942	5.0063
DCHSP70-36	DCHSP70-47	0.1235	3.0287	0.0408	11.8792
DCHSP70-37	DCHSP70-38	0.0185	0.1446	0.1280	1.7793
DCHSP70-41	DCHSP70-42	0.0458	0.1265	0.3617	4.4010
DCHSP70-45	DCHSP70-46	0.2545	0.6736	0.3777	24.4667
DCHSP70-48	DCHSP70-49	0.1030	0.1664	0.6189	9.9040

### Analysis of Protein-protein interaction (PPI)

**Fig. 7:**
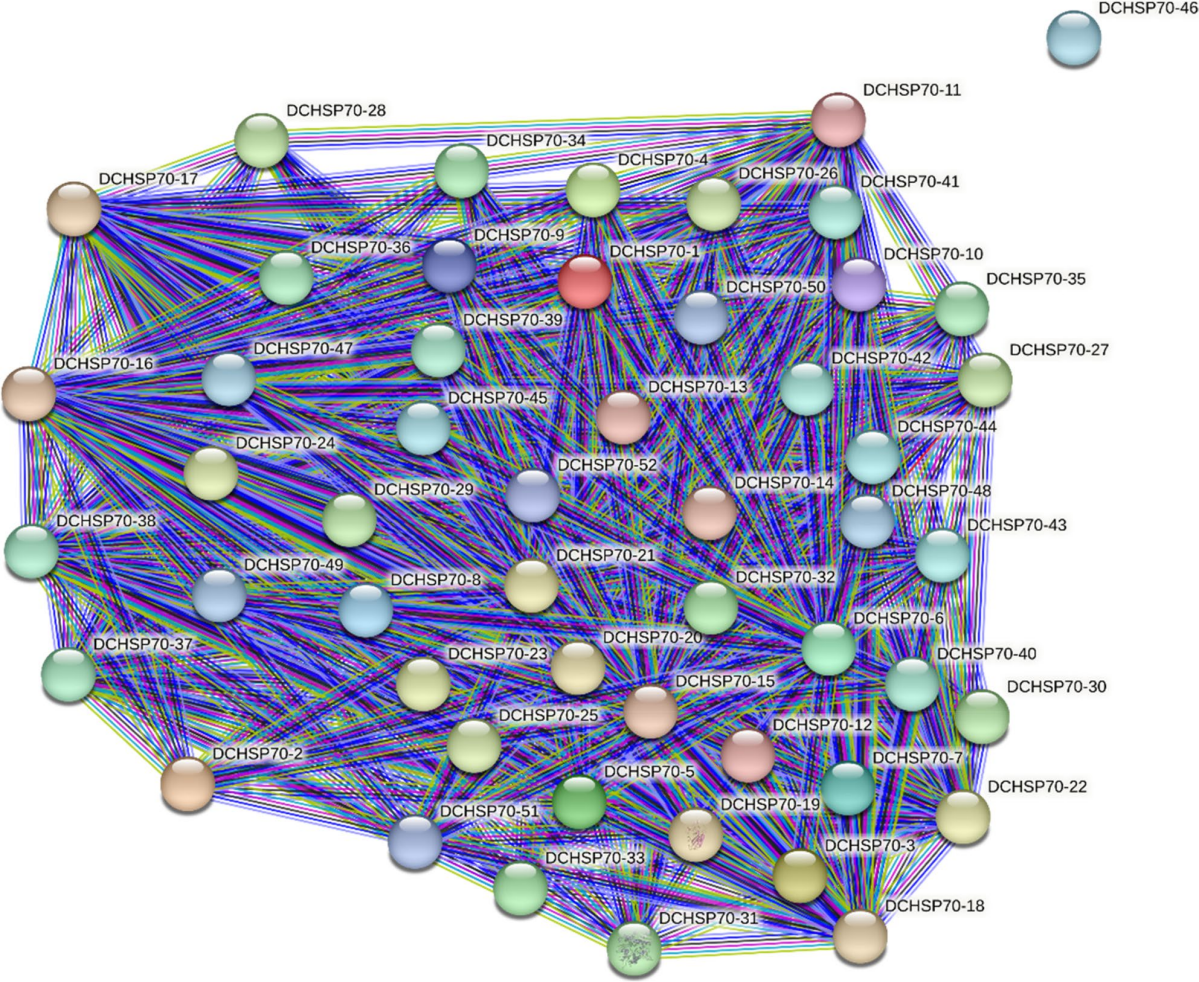
Protein-protein interaction (PPI) network of the DCHSP70 family. The network illustrates the predicted functional associations among the 52 DCHSP70 proteins

To predict the functional relationships and potential interaction networks among the DCHSP70 family members, a protein-protein interaction (PPI) network was constructed using the STRING database. The resulting network is composed of 52 nodes representing the DCHSP70 proteins and 571 edges representing the interactions between them (Fig. 7). The analysis revealed a highly interconnected network with an average node degree of 22, meaning that, on average, each DCHSP70 protein interacts with 22 other members of the family. The PPI enrichment p-value was < 1.0e-16, which is significantly lower than the expected number of edges (16) for a random set of proteins of similar size. This statistically significant enrichment indicates that the DCHSP70 proteins are not interacting at random; rather, they form a biologically cohesive cluster. This high degree of connectivity (average local clustering coefficient: 0.48) suggests that these family members likely function synergistically or as part of larger protein complexes to regulate stress responses and cellular homeostasis in *Daucus carota* [49].

### Gene ontology

**Fig. 8:**
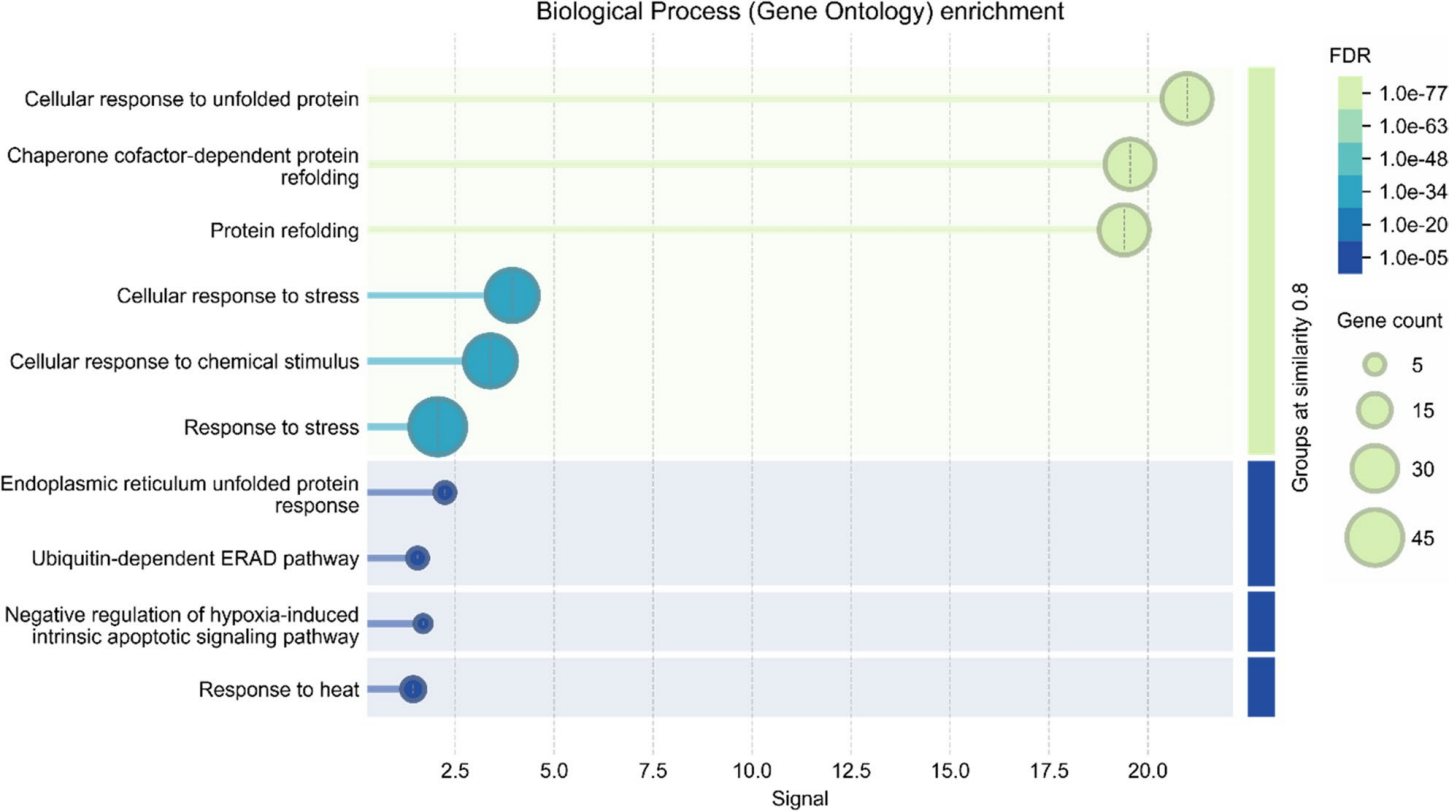
Gene Ontology (GO) enrichment analysis of biological processes for DCHSP70 proteins. the bar chart displays significantly enriched biological process terms. The x-axis represents the enrichment strength, while the color scale (right) indicates the False Discovery Rate (FDR) significance, ranging from blue (lower significance) to light green (higher significance). The size of the circles corresponds to the number of DCHSP70 genes associated with each specific term

The gene ontology enrichment analysis revealed a significant enrichment of terms related to protein quality control and stress tolerance **(**Fig. 8**)**. The most highly enriched biological processes “highest signal strength) were Cellular response to unfolded protein” and “Protein refolding” (1.0 × 10^− 63^), confirming the fundamental role of DCHSP70 proteins as molecular chaperones essential for maintaining protein homeostasis. Additionally, a substantial number of genes were associated with broader categories such as “Response to stress” and “cellular response to chemical stimulus,” highlighting the family’s extensive involvement in abiotic stress defense. Specific pathways, including the “Endoplasmic Reticulum Unfolded Protein Response” and Ubiquitin-dependent ERAD pathways, were also enriched, suggesting specialized functions for specific isoforms in ER stress response [50].

### Promoter analysis

**Fig. 9:**
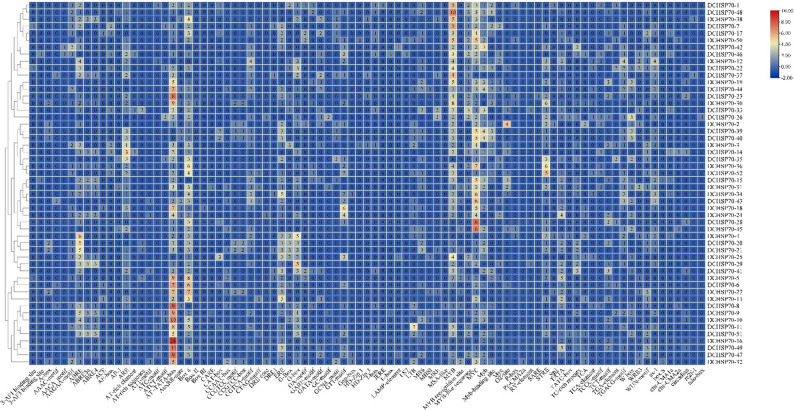
Cis-acting regulatory element analysis of the DCHSP70 gene promoters. The heatmap illustrates the number and distribution of identified cis elements in the 1,500 bp upstream regions of the 52 DCHSP70 genes. X-axis: Represents the different types of cis-regulatory elements (e.g., ABRE, LTR, MBS, TATA-box). Y-axis: Lists the 52 DCHSP70 genes, clustered based on the similarity of their promoter motif composition (dendrogram on the left). Color Scale: The color gradient from blue (low abundance/0) to red (high abundance/10+) indicates the frequency of each element within a specific promoter. High-frequency elements (red) signify strong regulatory potential for that specific pathway

**Fig. 10:**
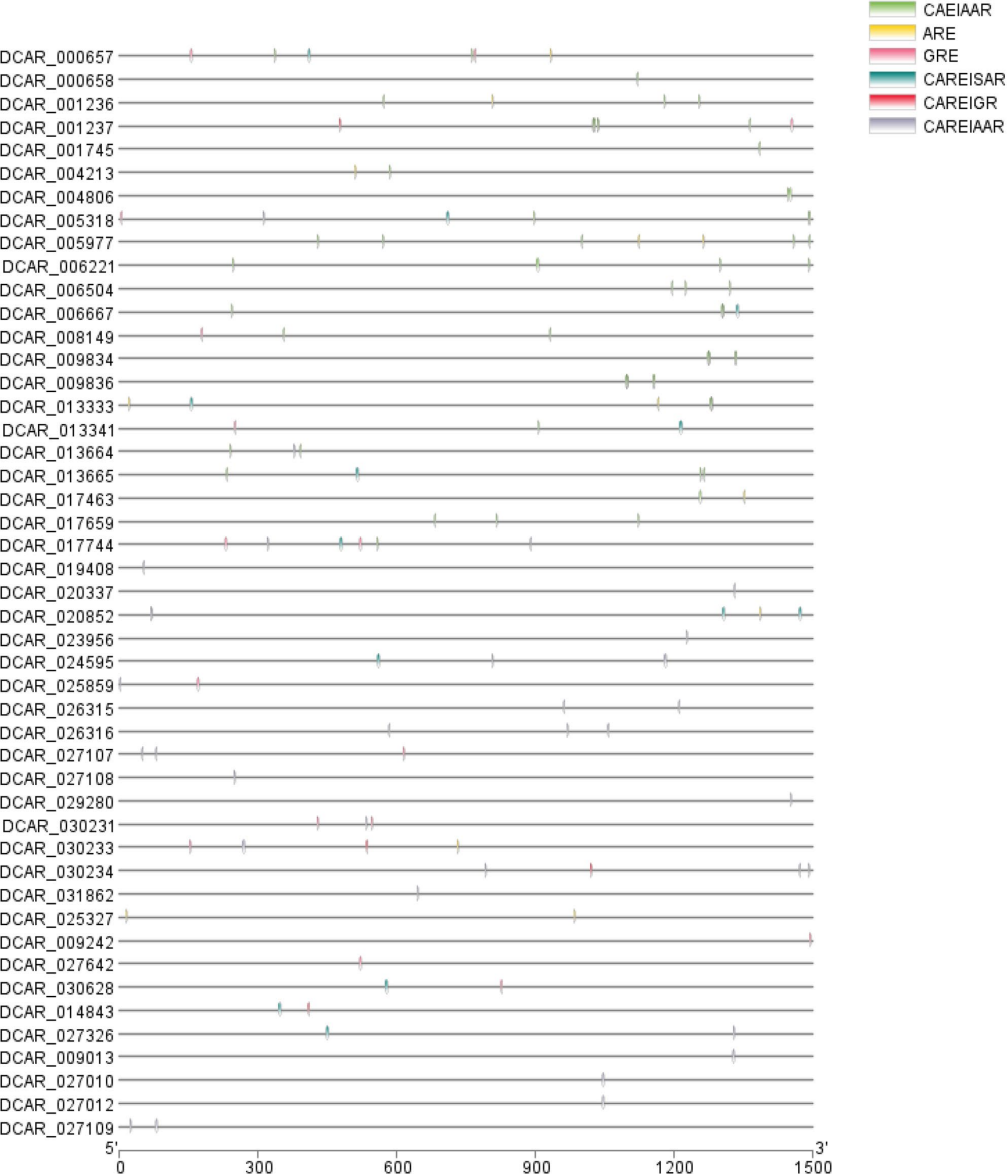
Distribution of hormone-responsive cis-regulatory elements in the promoter regions of DCHSP70 genes. The schematic diagram illustrates the specific locations of identified hormone-related motifs within the 1,500 bp upstream promoter sequences of DCHSP70 genes. The gray horizontal lines represent the promoter regions, while the colored markings indicate the positions of distinct cis-regulatory elements. The specific elements identified include ARE (Anaerobic Responsive Element), GRE, CAEIAAR, CAREISAR, CAREIGR, and CAREIAAR, as detailed in the legend

To gain insight into the transcriptional regulation of the DCHSP70 gene family, the 1500 bp upstream promoter regions of all 52 genes were analyzed (Figs. 9 and 10). A total of 4712 cis-regulatory elements were identified and classified into four major functional categories: promoter-related, light-responsive, hormone-responsive, and stress-responsive elements. Most of the identified motifs were promoter-related elements (e.g., TATA-box, CAAT-box), accounting for 78.57% (3,702 elements). This high abundance highlights the fundamental requirement for accurate transcriptional initiation across the gene family. Hormone-related motifs accounted for 4.73% (223 elements) of the total. ABRE (Abscisic acid-responsive element) was frequently observed, appearing as high-intensity (red/orange) blocks in specific genes (e.g., DCHSP70-20, DCHSP70-21), indicating their potential involvement in ABA signaling. Other significant motifs included the CGTCA-motif and TGACG-motif and the TGA-element (Auxin-responsive), suggesting that DCHSP70 genes are regulated by a complex hormonal network. Light-responsive elements (e.g., G-box, Box 4, GT1-motif) represent the second largest category (8.93%; 421 elements), indicating that light signaling plays a crucial role in DCHSP70 regulation. Furthermore, environmental stress-related elements (5.73%; 270 elements) such as LTR (Low-temperature responsiveness), MBS (MYB binding site involved in drought inducibility), and TC-rich repeats (defense and stress responsiveness) were unevenly distributed across the family (Supplementary File, Table 4).

### Structural characterization of DCHSP70 proteins

The three-dimensional (3D) structures of DCHSP70 proteins were predicted using homology modeling (Supplementary File, Table 5). Templates exhibiting high sequence identity ranging from 70% to 100% were used for model construction. The reliability of the predicted structures was assessed using Global Model Quality Estimation (GMQE) scores, which demonstrated high confidence values mostly > 0.80 [51, 52]. Furthermore, analysis of the quaternary structure indicated that these proteins are conserved as monomers in their physiological state (Supplementary File, Table 6). For DCHSP70-15 and DCHSP70-42, which lacked close homologs in the standard template library, models were generated using the threading-based I-TASSER server. DCHSP70-15 was modeled using template 5e84A, achieving a C-score of -2.18. DCHSP70-42 was modeled using template 7nh9A, with a C-score of -2.44 [47, 53] (Table 4).

**Table 4:** Structural modeling parameters for DCHSP70 proteins predicted using I-TASSER

Gene Name	Template	C-score	Estimated TM-score	Estimated RMSD
DCHSP70-15	5e84A	-2.18	0.46 ± 0.15	14.2 ± 3.8Å
DCHSP70-46	7nh9A	-2.44	0.43 ± 0.14	11.6 ± 4.5Å

## Discussion

The Heat Shock Protein 70 (HSP70) family is ubiquitous across the plant kingdom and plays a crucial role in maintaining cellular homeostasis under stress. While extensive studes have been conducted in model and crop species including *Arabidopsis thaliana* [24], barley [25], common bean [26], purple false brome *(Brachypodium distachyon)* [27], Cotton [28], Soybean (*Glycine max L*.) [29], tomato (*Solanum lycopersicum*) [3], Maize (*Zea mays*) [30], potato (*Solanum tuberosum L.*) [31], Pumpkin (*Cucurbita moschata*) [32] rice [33], *Theobroma cacao* [34], tobacco (*Nicotiana tabacum*) [35] but knowledge regarding this family in carrot is (*Daucus carota*) has remained limited. In this study, we performed a comprehensive genome-wide analysis, identifying 52 DCHSP70 genes. This number indicates a significant expansion of the family in the carrot compared to *Arabidopsis* (18 members), but is comparable to the numbers found in larger genomes like soybean (61 members) [54, 55]. Subcellular localization analysis revealed that the majority of DCHSP70 proteins (29 members) are predicted to function in the cytoplasm. This cytoplasmic count is notably higher than that of *Arabidopsis* (5 members) [24], and rice (11 members) [33], and approaches the number found in soybean (34 members) [56]. The expansion of cytoplasmic isoforms in carrots suggests a specialized evolutionary adaptation.

The integration of phylogenetic analysis with gene structure and motif composition revealed a distant pattern of evolutionary conservation. Members cluster within the same phylogenetic subgroups, particularly regarding structural variations that were observed between different subgroups, particularly regarding intron lengths and phases. These structural divergences likely reflect the evolutionary history of the family. The introduction of introns or loss events has contributed to the functional diversification of specific isoforms. The conservation of motif order within subgroups further supports the reliability of our phylogenetic classification and suggests that these motifs are essential for group-specific functions.

Gene duplication is a primary driving force in plant genome evolution, often leading to gene family expansion and neofunctionalization [57]. While duplication can occur via segmental, tandem, or whole-genome events [58]. Our synteny analysis indicates that segmental duplication has played a predominant role in the evolution of the DCHSP70 family. We identified 21 paralogous pairs derived from segmental duplication events. The retention of these duplicated genes, coupled with the purifying selection pressure observed in our Ka/Ks analysis, suggests that these gene pairs are functionally important and have been maintained to reinforce the plant’s stress tolerance mechanisms.

The predicted protein-protein interaction (PPI) network provides theoretical insight into the physiological roles of DCHSP70s. The dense connectivity suggests that these proteins do not function in isolation but rather form homo-or heterodimers to participate in complex metabolic networks and signal transduction pathways.

Furthermore, the analysis of cis-regulatory elements in the promoter regions highlights the multi-functional nature of DCHSP70 genes. Beyond the core promoter elements (78% of identified motifs), we detected a significant presence of stress-responsive motifs. Notably, the presence of Heat Shock Factors (HSFs) to regulate thermal stress responses [59, 60], while DREs govern responses to cold and drought [53]. The enrichment of these elements in DCHSP70 promoters strongly suggests that this gene family is a critical component of the carrots’ defense system against abiotic stress.

In summary, the DCHSP70 family in carrots has undergone significant expansion, primarily through segmental duplication, resulting in a diverse set of genes equipped with specialized regulatory elements. These findings not only advance our understanding of the DCHSP70 family but also provide valuable genetic targets for breeding programs aimed at improving stress tolerance in carrot and related Apicaceae crops.

## Conclusions

This study identified 52 HSP70 genes in the Daucus carota genome, revealing that segmental duplication has been the primary driver of the family’s evolutionary expansion. Phylogenetic and structural analysis demonstrated high conservation across the family, while the abundance of stress-responsive cis-regulatory elements in promoter regions highlights their pivotal role in abiotic stress adaptation. These findings provide a foundational resource for future functional genomics and molecular breeding efforts aimed at improving stress resilience in carrots.

## Supplementary Information

Supplementary Material 1.

Supplementary Material 2.

Supplementary Material 3.

Supplementary Material 4.

Supplementary Material 5.

Supplementary Material 6.

## Data Availability

All the required data is provided here; however, further relevant data/information, the corresponding author may be contacted.

## References

[1] Carmo-Silva AE, et al. Decreased CO2 availability and inactivation of Rubisco limit photosynthesis in cotton plants under heat and drought stress in the field. Environ Exp Bot. 2012;83:1–11.

[2] Awasthi R, et al. Individual and combined effects of transient drought and heat stress on carbon assimilation and seed filling in Chickpea. Funct Plant Biol. 2014;41(11):1148–67. 32481065 10.1071/FP13340

[3] Ahmad A, Diwan H, Abrol YP. Global climate change, stress and plant productivity. Abiotic stress adaptation in plants: physiological, molecular and genomic foundation. 2010. pp. 503–521.

[4] Maestri E, et al. Molecular genetics of heat tolerance and heat shock proteins in cereals. Plant Mol Biol. 2002;48:667–81. 11999842 10.1023/a:1014826730024

[5] Ruan Y-L, et al. Sugar input, metabolism, and signaling mediated by invertase: roles in development, yield potential, and response to drought and heat. Mol Plant. 2010;3(6):942–55. 20729475 10.1093/mp/ssq044

[6] Xu Z-S, et al. Heat shock protein 90 in plants: molecular mechanisms and roles in stress responses. Int J Mol Sci. 2012;13(12):15706–23. 23443089 10.3390/ijms131215706 PMC3546657

[7] Qin D, et al. Heat stress-responsive transcriptome analysis in heat susceptible and tolerant wheat (Triticum aestivum L.) by using wheat genome array. BMC Genomics. 2008;9:1–19. 18808683 10.1186/1471-2164-9-432 PMC2614437

[8] Larkindale J, Vierling E. Core genome responses involved in acclimation to high temperature. Plant Physiol. 2008;146(2):748. 18055584 10.1104/pp.107.112060 PMC2245833

[9] Tissiéres A, Mitchell HK, Tracy UM. Protein synthesis in salivary glands of drosophila melanogaster: relation to chromosome puffs. J Mol Biol. 1974;84(3):389–98. 4219221 10.1016/0022-2836(74)90447-1

[10] Lee DG, et al. A proteomic approach in analyzing heat-responsive proteins in rice leaves. Proteomics. 2007;7(18):3369–83. 17722143 10.1002/pmic.200700266

[11] Queitsch C, et al. Heat shock protein 101 plays a crucial role in thermotolerance in Arabidopsis. Plant Cell. 2000;12(4):479–92. 10760238 10.1105/tpc.12.4.479 PMC139847

[12] Young JC, Moarefi I, Hartl FU. Hsp90: a specialized but essential protein-folding tool. J Cell Biol. 2001;154(2):267. 11470816 10.1083/jcb.200104079 PMC2150759

[13] Pratt WB, Krishna P, Olsen LJ. Hsp90-binding Immunophilins in plants: the protein movers. Trends Plant Sci. 2001;6(2):54–8. 11173288 10.1016/s1360-1385(00)01843-4

[14] Queitsch C, Sangster TA, Lindquist S. Hsp90 as a capacitor of phenotypic variation. Nature. 2002;417(6889):618–24. 12050657 10.1038/nature749

[15] Bösl B, Grimminger V, Walter S. The molecular chaperone Hsp104—a molecular machine for protein disaggregation. J Struct Biol. 2006;156(1):139–48. 16563798 10.1016/j.jsb.2006.02.004

[16] Glover JR, Lindquist S. Hsp104, Hsp70, and Hsp40: a novel chaperone system that rescues previously aggregated proteins. Cell. 1998;94(1):73–82. 9674429 10.1016/s0092-8674(00)81223-4

[17] Goloubinoff P, et al. Sequential mechanism of solubilization and refolding of stable protein aggregates by a bichaperone network. Proc Natl Acad Sci. 1999;96(24):13732–7. 10570141 10.1073/pnas.96.24.13732 PMC24133

[18] Zhu X, et al. Structural analysis of substrate binding by the molecular chaperone DnaK. Science. 1996;272(5268):1606–14. 8658133 10.1126/science.272.5268.1606 PMC5629921

[19] Masand S, Yadav SK. Overexpression of MuHSP70 gene from macrotyloma uniflorum confers multiple abiotic stress tolerance in Transgenic Arabidopsis Thaliana. Mol Biol Rep. 2016;43:53–64. 26694324 10.1007/s11033-015-3938-y

[20] Hartl FU. Molecular chaperones in cellular protein folding. Nature. 1996;381(6583):571–80. 8637592 10.1038/381571a0

[21] Frydman J. Folding of newly translated proteins in vivo: the role of molecular chaperones. Annu Rev Biochem. 2001;70(1):603–47. 11395418 10.1146/annurev.biochem.70.1.603

[22] Kim BH, Schöffl F. Interaction between Arabidopsis heat shock transcription factor 1 and 70 kDa heat shock proteins. J Exp Bot. 2002;53(367):371–5. 11807141 10.1093/jexbot/53.367.371

[23] Iorizzo M, et al. Genetic structure and domestication of Carrot (Daucus Carota subsp. sativus)(Apiaceae). Am J Bot. 2013;100(5):930–8. 23594914 10.3732/ajb.1300055

[24] Lin B-L, et al. Genomic analysis of the Hsp70 superfamily in Arabidopsis Thaliana. Cell stress & chaperones; 2001;6:201-3. 10.1379/1466-1268(2001)006<0201:gaoths>2.0.co;2 PMC434401 11599561

[25] Chaudhary R, et al. Genome-wide identification and expression analysis of Hsp70, Hsp90, and Hsp100 heat shock protein genes in barley under stress conditions and reproductive development. Funct Integr Genom. 2019;19:1007–22. 10.1007/s10142-019-00695-y 31359217

[26] Büyük İ, et al. Genome-wide identification of salinity responsive HSP70 s in common bean. Mol Biol Rep. 2016;43:1251–66. 27558093 10.1007/s11033-016-4057-0

[27] Wen F, et al. Genome-wide survey of heat shock factors and heat shock protein 70s and their regulatory network under abiotic stresses in brachypodium distachyon. PLoS ONE. 2017;12(7):e0180352. 28683139 10.1371/journal.pone.0180352 PMC5500289

[28] Rehman A, et al. Genome-wide identification and characterization of HSP70 gene family in four species of cotton. Genomics. 2020;112(6):4442–53. 32739432 10.1016/j.ygeno.2020.07.039

[29] Zhang L, et al. Genome-wide analysis and expression profiling under heat and drought treatments of HSP70 gene family in soybean (Glycine max L). Front Plant Sci. 2015;6:773. 26442082 10.3389/fpls.2015.00773 PMC4585176

[30] Jiang L, et al. Genome-wide identification, classification and expression analysis of the Hsf and Hsp70 gene families in maize. Gene. 2021;770:145348. 33333230 10.1016/j.gene.2020.145348

[31] Liu J, et al. The Hsp70 gene family in solanum tuberosum: genome-wide identification, phylogeny, and expression patterns. Sci Rep. 2018;8(1):16628. 30413778 10.1038/s41598-018-34878-7 PMC6226454

[32] Chen C, et al. TBtools: an integrative toolkit developed for interactive analyses of big biological data. Mol Plant. 2020;13(8):1194–202. 32585190 10.1016/j.molp.2020.06.009

[33] Jung K-H, et al. Genome-wide expression analysis of HSP70 family genes in rice and identification of a cytosolic HSP70 gene highly induced under heat stress. Funct Integr Genom. 2013;13:391–402. 10.1007/s10142-013-0331-6 23852542

[34] La V. Genome-Wide identification and analysis of heat shock protein 70 family in theobroma Cacao. Pakistan J Biol Sciences: PJBS. 2022;25(7):608–18. 10.3923/pjbs.2022.608.618 36098167

[35] Song Z, et al. Genome-wide identification and characterization of Hsp70 gene family in Nicotiana tabacum. Mol Biol Rep. 2019;46(2):1941–54. 30710231 10.1007/s11033-019-04644-7

[36] Goodstein DM, et al. Phytozome: a comparative platform for green plant genomics. Nucleic Acids Res. 2012;40(D1):D1178–86. 22110026 10.1093/nar/gkr944 PMC3245001

[37] Gasteiger E, et al. ExPASy: the proteomics server for in-depth protein knowledge and analysis. Nucleic Acids Res. 2003;31(13):3784–8. 12824418 10.1093/nar/gkg563 PMC168970

[38] Kumar S, et al. MEGA12: Molecular Evolutionary Genetic Analysis version 12 for adaptive and green computing. Mol Biol Evol. 2024;41(12):msae263. 10.1093/molbev/msae263 PMC11683415 39708372

[39] Letunic I, Bork P. Interactive tree of life (iTOL) v5: an online tool for phylogenetic tree display and annotation. Nucleic Acids Res. 2021;49(W1):W293–6. 33885785 10.1093/nar/gkab301 PMC8265157

[40] Lu S, et al. CDD/SPARCLE: the conserved domain database in 2020. Nucleic Acids Res. 2020;48(D1):D265–8. 31777944 10.1093/nar/gkz991 PMC6943070

[41] Bailey TL, et al. MEME: discovering and analyzing DNA and protein sequence motifs. Nucleic Acids Res. 2006;34(suppl2):W369–73. 16845028 10.1093/nar/gkl198 PMC1538909

[42] Song X, et al. Deciphering the high-quality genome sequence of coriander that causes controversial feelings. Plant Biotechnol J. 2020;18(6):1444–56. 31799788 10.1111/pbi.13310 PMC7206992

[43] Wu X-J, et al. Genome-wide analysis of PHD family transcription factors in Carrot (Daucus Carota L.) reveals evolution and response to abiotic stress. Acta Physiol Plant. 2016;38:1–15.

[44] Lescot M, et al. PlantCARE, a database of plant cis-acting regulatory elements and a portal to tools for in Silico analysis of promoter sequences. Nucleic Acids Res. 2002;30(1):325–7. 11752327 10.1093/nar/30.1.325 PMC99092

[45] Lescot M, et al. PlantCARE, a database of plant cis-acting regulatory elements and a portal to tools for in silico analysis of promoter sequences. Nucleic Acids Res. 2002;30(1):325–7. 11752327 10.1093/nar/30.1.325 PMC99092

[46] Bertoni M, et al. Modeling protein quaternary structure of homo-and hetero-oligomers beyond binary interactions by homology. Sci Rep. 2017;7(1):10480. 28874689 10.1038/s41598-017-09654-8 PMC5585393

[47] Zhang Y. I-TASSER server for protein 3D structure prediction. BMC Bioinformatics. 2008;9(1):40. 18215316 10.1186/1471-2105-9-40 PMC2245901

[48] Hurst LD. The Ka/Ks ratio: diagnosing the form of sequence evolution. Trends Genet. 2002;18(9):486–7. 12175810 10.1016/s0168-9525(02)02722-1

[49] Wang W, et al. Role of plant heat-shock proteins and molecular chaperones in the abiotic stress response. Trends Plant Sci. 2004;9(5):244–52. 15130550 10.1016/j.tplants.2004.03.006

[50] Wang W, et al. Role of plant heat-shock proteins and molecular chaperones in the abiotic stress response. Trends Plant Sci. 2004;9(5):244–52. 15130550 10.1016/j.tplants.2004.03.006

[51] Waterhouse A, et al. SWISS-MODEL: homology modelling of protein structures and complexes. Nucleic Acids Res. 2018;46(W1):W296–303. 29788355 10.1093/nar/gky427 PMC6030848

[52] Benkert P, Biasini M, Schwede T. Toward Estimation Absolute Qual Individual Protein Struct Models Bioinf. 2011;27(3):343–50. 10.1093/bioinformatics/btq662 PMC3031035 21134891

[53] Yang J, et al. The I-TASSER suite: protein structure and function prediction. Nat Methods. 2015;12(1):7–8. 25549265 10.1038/nmeth.3213 PMC4428668

[54] Cho EK, Choi YJ. A nuclear-localized HSP70 confers thermoprotective activity and drought-stress tolerance on plants. Biotechnol Lett. 2009;31:597–606. 19034388 10.1007/s10529-008-9880-5

[55] Kose S, Furuta M, Imamoto N. Hikeshi, a nuclear import carrier for Hsp70s, protects cells from heat shock-induced nuclear damage. Cell. 2012;149(3):578–89. 22541429 10.1016/j.cell.2012.02.058

[56] Sarkar NK, Kundnani P, Grover A. Functional analysis of Hsp70 superfamily proteins of rice (Oryza sativa). Cell Stress Chaperones. 2013;18(4):427–37. 23264228 10.1007/s12192-012-0395-6 PMC3682022

[57] Lespinet O, et al. The role of lineage-specific gene family expansion in the evolution of eukaryotes. Genome Res. 2002;12(7):1048–59. 12097341 10.1101/gr.174302 PMC186617

[58] Sémon M, Wolfe KH. Consequences of genome duplication. Curr Opin Genet Dev. 2007;17(6):505–12. 18006297 10.1016/j.gde.2007.09.007

[59] Sung DY, Kaplan F, Guy CL. Plant Hsp70 molecular chaperones: protein structure, gene family, expression and function. Physiol Plant. 2001;113(4):443–51.

[60] Czarnecka E, Key JL, Gurley WB. Regulatory domains of the Gmhsp17. 5-E heat shock promoter of soybean. Mol Cell Biol. 1989;9(8):3457–63. 2796991 10.1128/mcb.9.8.3457-3463.1989 PMC362392

