# Supplementary material for: In silico characterization, evolutionary analysis, and structural modeling of HSP70 gene family in carrot (Daucus Carota L.)
Source: BMC Plant Biol. 2026 Jan 15;26:272. doi: 10.1186/s12870-026-08102-y (PMC12892451; doi:10.1186/s12870-026-08102-y)
Supplement: Supplementary file 6 — Supplementary Material 6. [file 12870_2026_8102_MOESM6_ESM.docx]

| **3D Structure for HSP70 in *Daucus carota*** | | | |
| --- | --- | --- | --- |
| 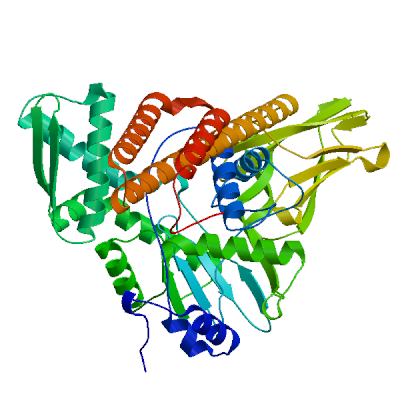 | 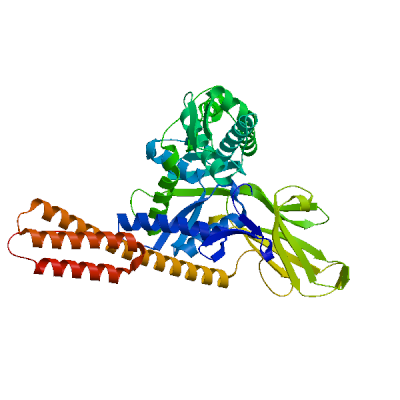 | 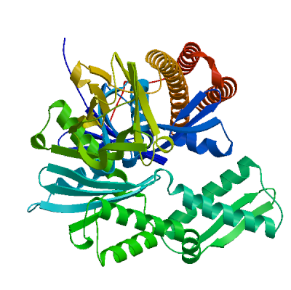 | 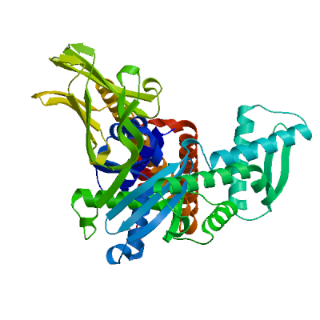 |
| DCHSP70-1 | DCHSP70-2 | DCHSP70-3 | DCHSP70-4 |
|  |  |  |  |
| 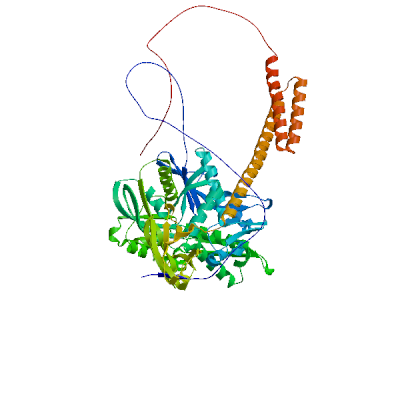 | 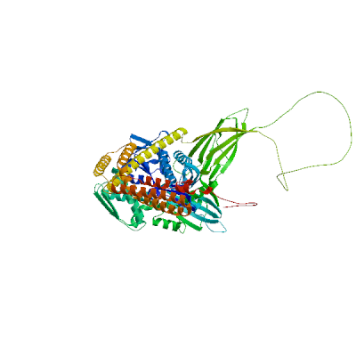 | 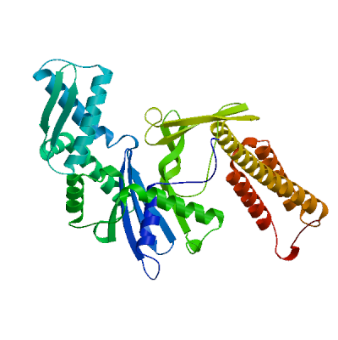 | 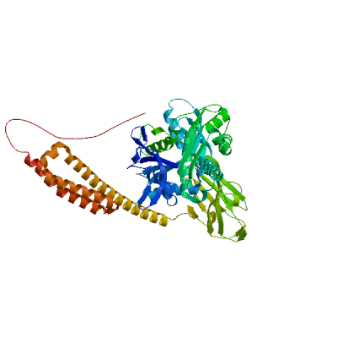 |
| DCHSP70-5 | DCHSP70-6 | DCHSP70-7 | DCHSP70-8 |
|  |  |  |  |
| 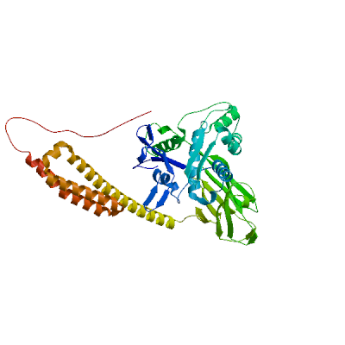 | 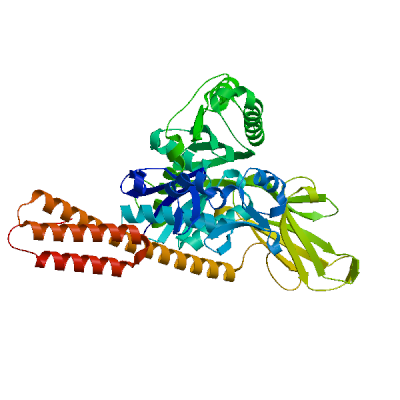 | 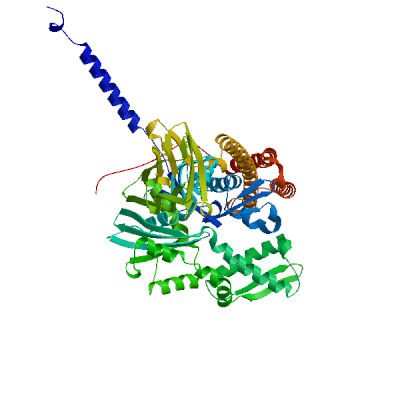 | 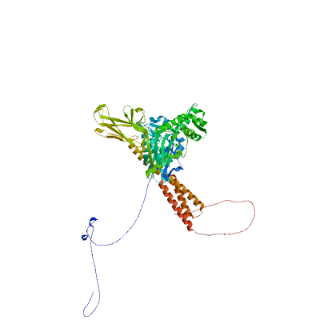 |
| DCHSP70-9 | DCHSP70-10 | DCHSP70-11 | DCHSP70-12 |
|  |  |  |  |
| 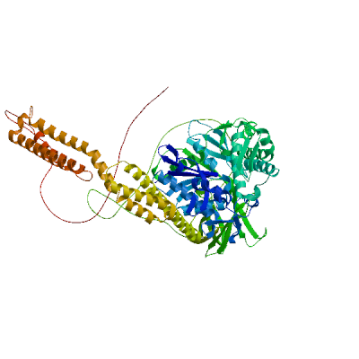 | 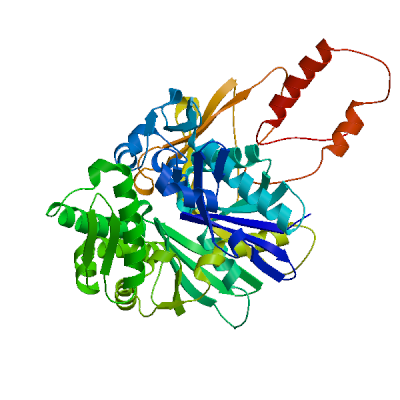 | 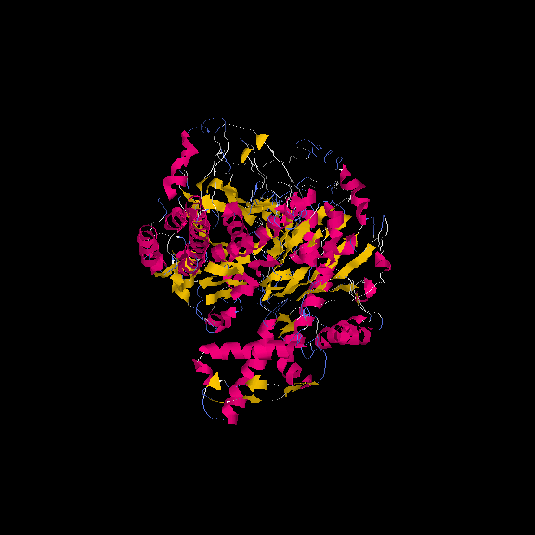 | 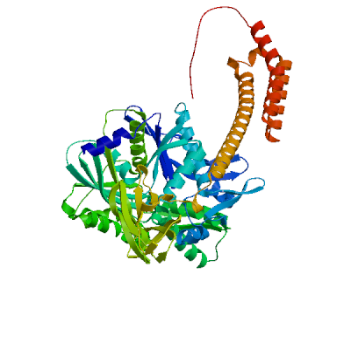 |
| DCHSP70-13 | DCHSP70-14 | DCHSP70-15 | DCHSP70-16 |
|  |  |  |  |
| 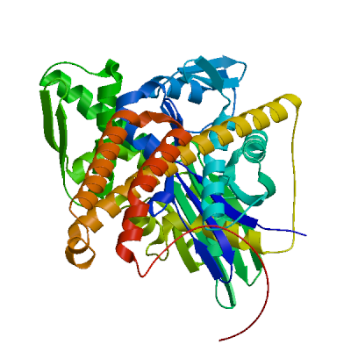 | 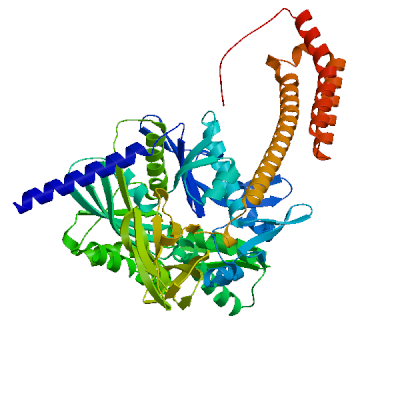 | 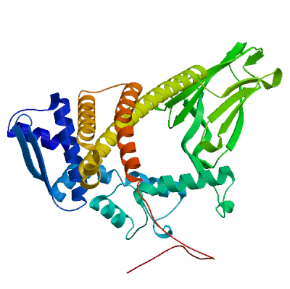 | 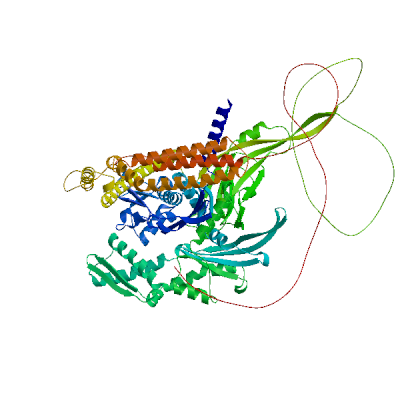 |
| DCHSP70-17 | DCHSP70-18 | DCHSP70-19 | DCHSP70-20 |
|  |  |  |  |
| 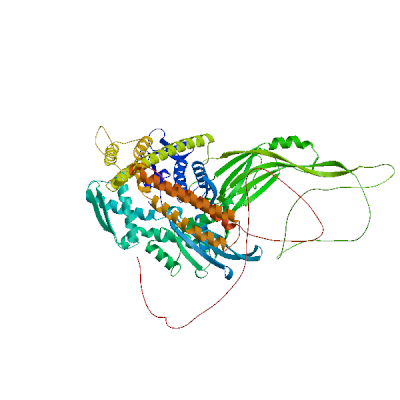 | 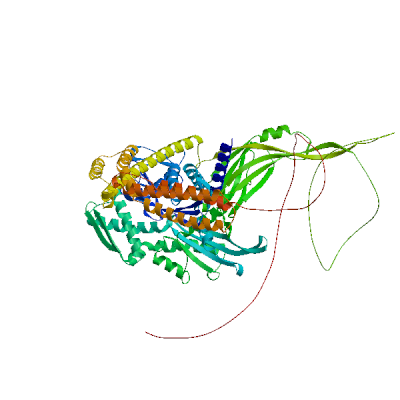 | 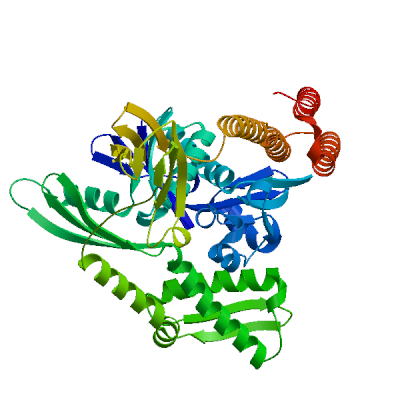 | 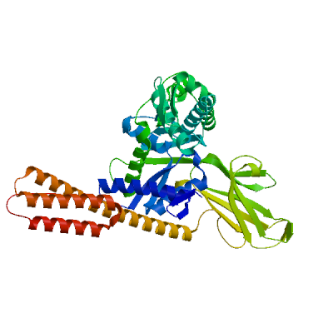 |
| DCHSP70-21 | DCHSP70-22 | DCHSP70-23 | DCHSP70-24 |
|  |  |  |  |
| 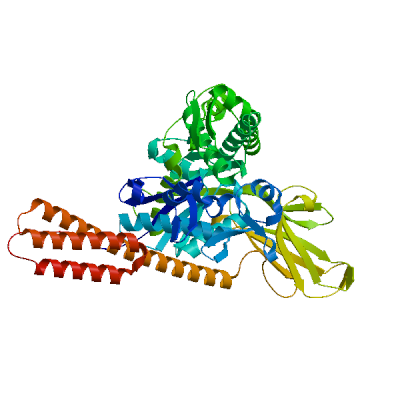 | 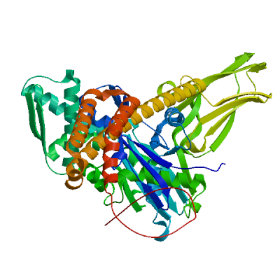 | 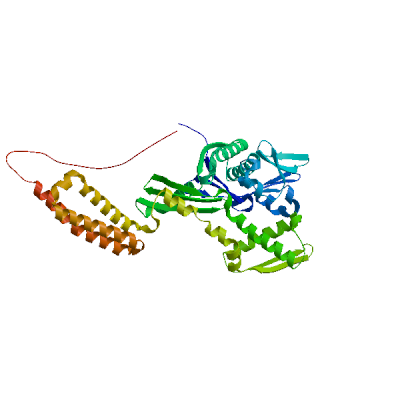 | 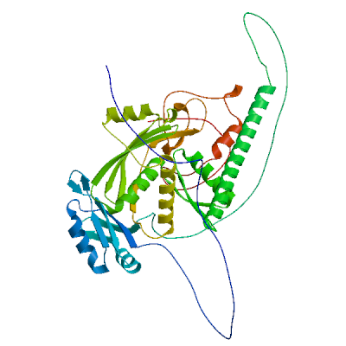 |
| DCHSP70-25 | DCHSP70-26 | DCHSP70-27 | DCHSP70-28 |
|  |  |  |  |
| 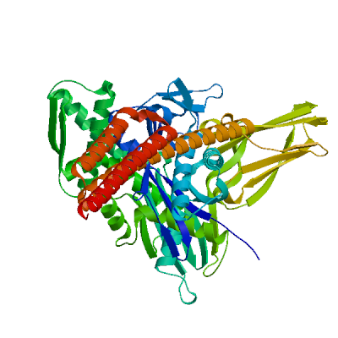 | 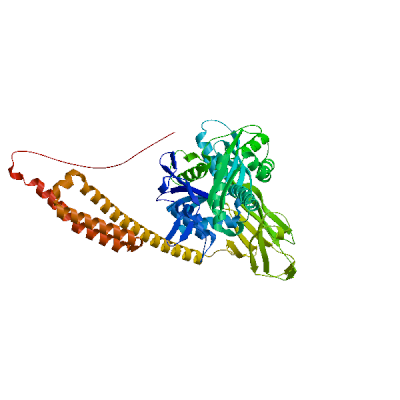 | 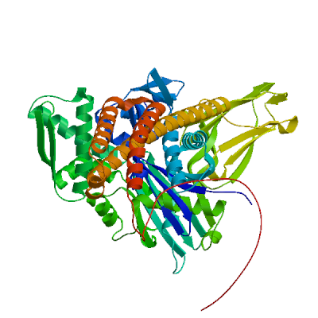 | 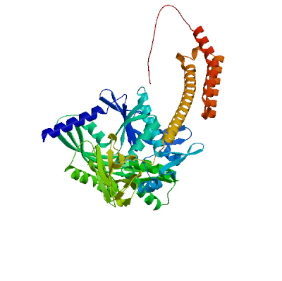 |
| DCHSP70-29 | DCHSP70-30 | DCHSP70-31 | DCHSP70-32 |
|  |  |  |  |
| 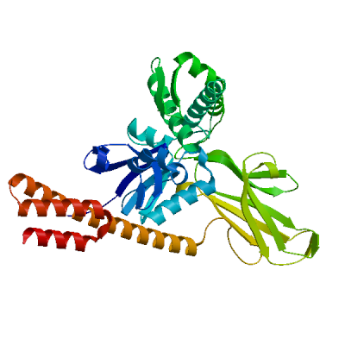 | 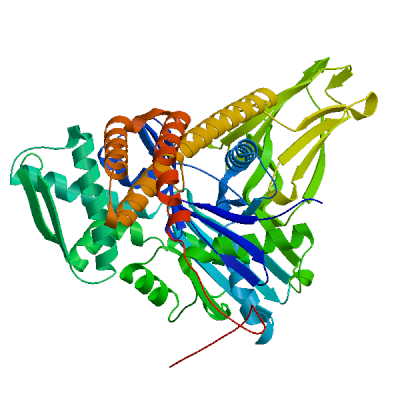 | 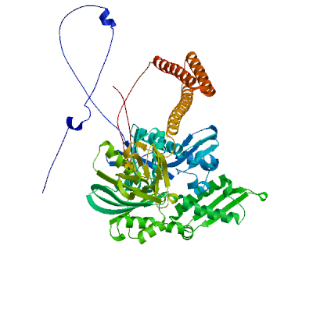 | 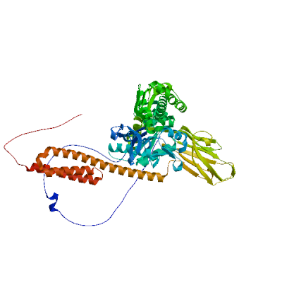 |
| DCHSP70-33 | DCHSP70-34 | DCHSP70-35 | DCHSP70-36 |
|  |  |  |  |
| 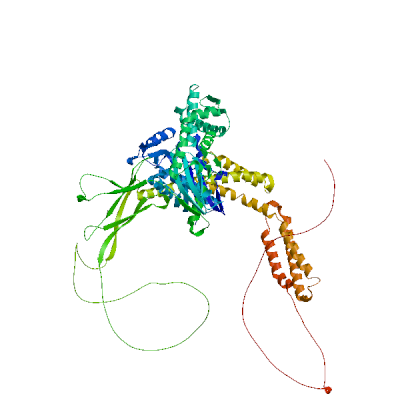 | 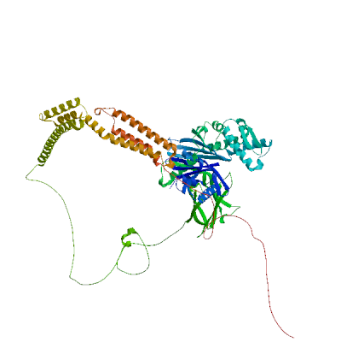 | 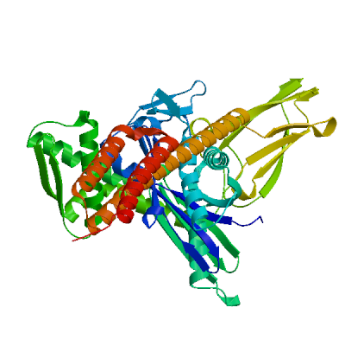 | 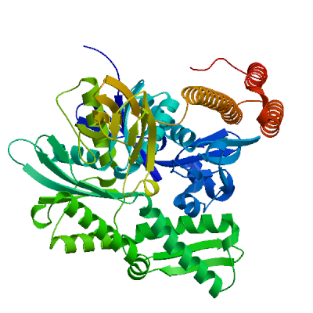 |
| DCHSP70-37 | DCHSP70-38 | DCHSP70-39 | DCHSP70-40 |
|  |  |  |  |
| 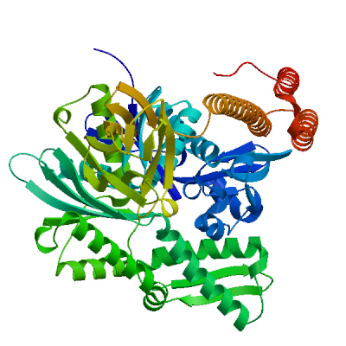 | 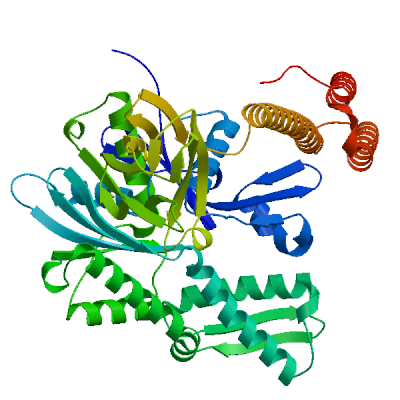 | 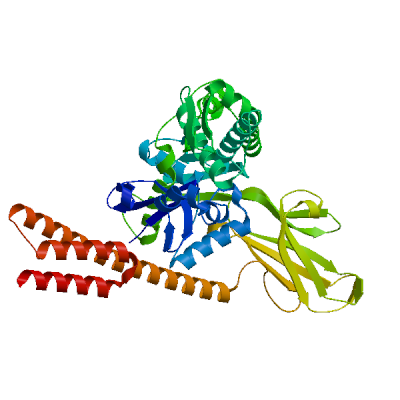 | 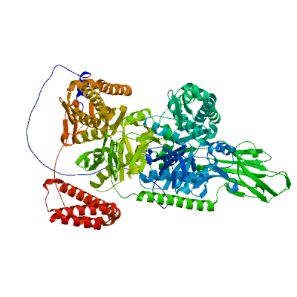 |
| DCHSP70-41 | DCHSP70-42 | DCHSP70-43 | DCHSP70-44 |
|  |  |  |  |
| 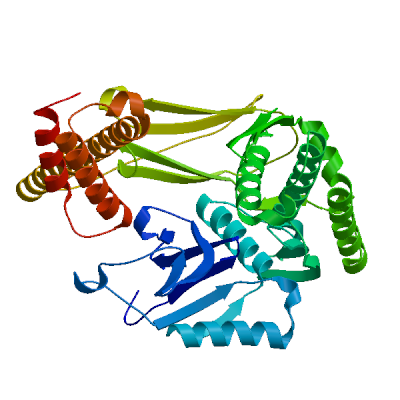 | 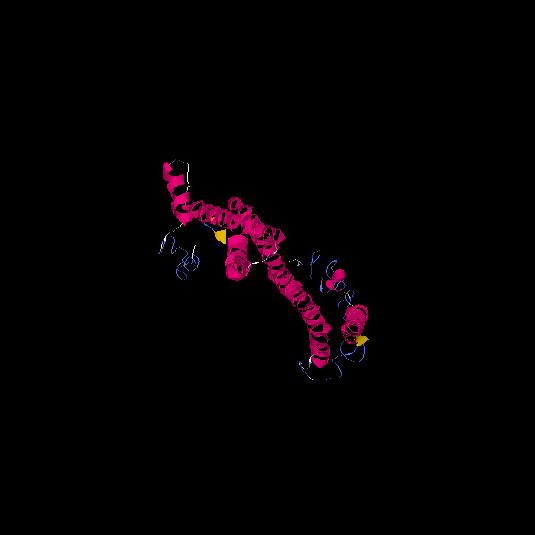 | 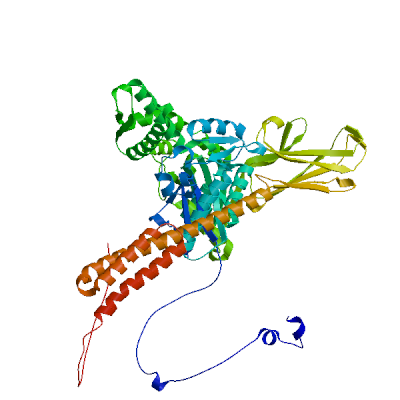 | 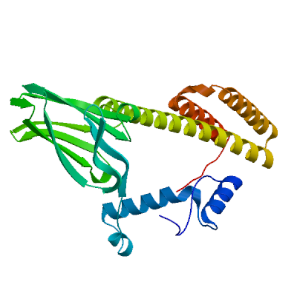 |
| DCHSP70-45 | DCHSP70-46 | DCHSP70-47 | DCHSP70-48 |
|  |  |  |  |
| 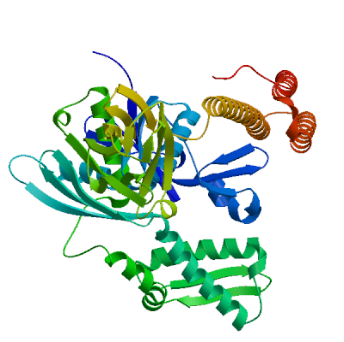 | 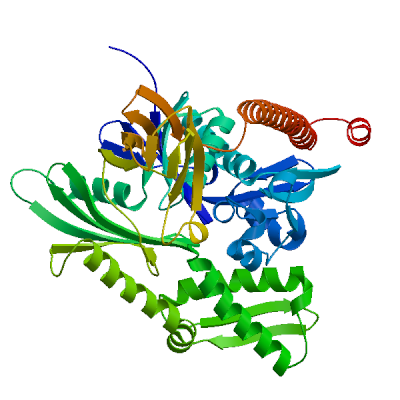 | 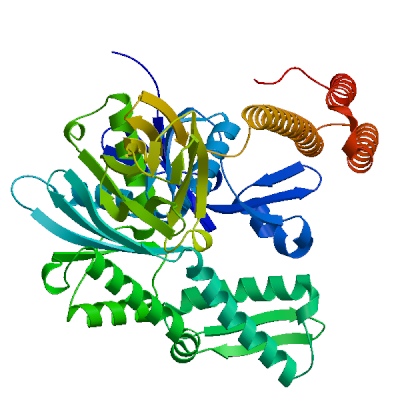 | 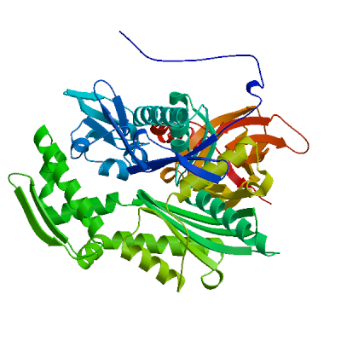 |
| DCHSP70-49 | DCHSP70-50 | DCHSP70-51 | DCHSP70-52 |
|  |  |  |  |
